# Available energy fluxes drive a transition in the diversity, stability, and functional structure of microbial communities

**DOI:** 10.1371/journal.pcbi.1006793

**Published:** 2019-02-05

**Authors:** Robert Marsland, Wenping Cui, Joshua Goldford, Alvaro Sanchez, Kirill Korolev, Pankaj Mehta

**Affiliations:** 1 Department of Physics, Boston University, Boston, MA, USA; 2 Department of Physics, Boston College, Chestnut Hill, MA, USA; 3 Bioinformatics Program, Boston University, Boston, MA, USA; 4 Department of Ecology and Evolutionary Biology, Yale University, New Haven, CT, USA; Rutgers University, UNITED STATES

## Abstract

A fundamental goal of microbial ecology is to understand what determines the diversity, stability, and structure of microbial ecosystems. The microbial context poses special conceptual challenges because of the strong mutual influences between the microbes and their chemical environment through the consumption and production of metabolites. By analyzing a generalized consumer resource model that explicitly includes cross-feeding, stochastic colonization, and thermodynamics, we show that complex microbial communities generically exhibit a transition as a function of available energy fluxes from a “resource-limited” regime where community structure and stability is shaped by energetic and metabolic considerations to a diverse regime where the dominant force shaping microbial communities is the overlap between species’ consumption preferences. These two regimes have distinct species abundance patterns, different functional profiles, and respond differently to environmental perturbations. Our model reproduces large-scale ecological patterns observed across multiple experimental settings such as nestedness and differential beta diversity patterns along energy gradients. We discuss the experimental implications of our results and possible connections with disorder-induced phase transitions in statistical physics.

## Author summary

The diversity, stability and functional structure of microbial communities have dramatic effects on the health of humans and of ecosystems. The complexity of these communities has so far precluded the development of a general predictive model that would capture the dependence of these features on environmental conditions. We confronted this challenge by constructing a flexible simulation framework, and randomly sampling parameters under a variety of modeling assumptions to identify generic patterns. We found two qualitatively distinct regimes of community structure, which reproduce observed patterns of biodiversity, and make new predictions about stability and function.

## Introduction

Microbial communities inhabit every corner of our planet, from our own nutrient-rich guts to the remote depths of the ocean floor. Different environments harbor very different levels of microbial diversity: in some samples of non-saline water at mild temperature and pH, nearly 3,000 coexisting types of bacteria can be detected, whereas at ambient temperatures warmer than 40° C, most cataloged samples contain fewer than 100 distinct variants [1]. The functional structure of these communities is also highly variable, with functional traits often reflecting the environment in which the communities are found [1, 2]. A central goal of microbial community ecology is to understand how these variations in diversity, stability and functional structure [3] arise from an interplay of environmental factors such as energy and resource availability [4, 5] and ecological processes such as competition [6–9] and stochastic colonization [10–13].

This endeavor is complicated by the fact that microbes dramatically modify their abiotic environments through consumption and secretion of organic and inorganic compounds. This happens on a global scale, as in the Great Oxidation Event two billion years ago [14, 15], and also on smaller scales relevant to agriculture, industry and medicine. In this sense, every microbe is an “ecosystem engineer” [16]. Metabolic modeling and experiments suggests that metabolically mediated syntrophic interactions should be a generic feature of microbial ecology [17–19] and that complex microbial communities can self-organize even in constant environments with no spatial structure or predation [17, 20]. For these reasons, there has been significant interest in developing new models for community assembly suited to the microbial setting [21–25].

Here, we present a statistical physics-inspired consumer resource model for microbial community assembly that builds upon the simple model introduced in [17] and explicitly includes energetic fluxes, stochastic colonization, syntrophy, and resource competition. We focus on modeling complex communities with many species and metabolites. By necessity, any mathematical model of such a large, diverse ecosystem will contain thousands of parameters that are hard to measure. To circumvent this problem, we take a statistical physics approach where all consumer preferences and metabolic parameters are drawn from random distributions.

This approach to modeling complex systems has its root in the pioneering work of Wigner on the spectrum of heavy nuclei [26] and was adapted by May to ecological settings [27]. Recently, there has been a renewed interest in using these ideas to understand complex systems in both many-body physics (reviewed in [28]) and community assembly [12, 17, 25, 29–35]. The key insight underlying this approach is that generic and reproducible large-scale patterns observed across multiple settings likely reflect *typical* properties, rather than fine tuned features of any particular realization or community. Consistent with this idea, it was recently shown that a generalized consumer resource model with random parameters can reproduce many of the patterns observed in experiments where natural communities were grown in synthetic minimal environments [17].

In this paper, we ask how varying the energy flux into an ecosystem and the amount of cross-feeding affects microbial community assembly. We find that the resulting communities generically fall into two distinct regimes, characterized by qualitative differences in their community-level metabolic networks, functional structures, responses to environmental perturbations, and large-scale biodiversity patterns. We show our model predictions are consistent with data from the Tara Oceans database [36] and the Earth Microbiome Project [1], and propose feasible experimental tests using synthetic communities.

## Methods

**Fig 1:**
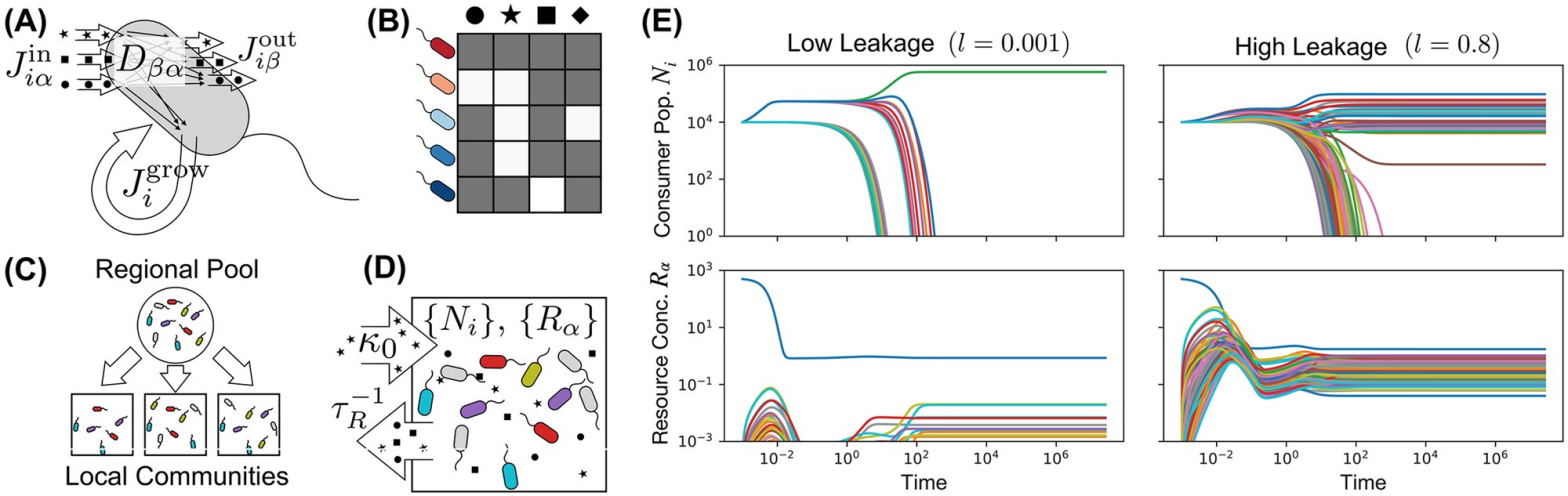
Microbial communities engineer complex chemical environments using a single energy source. (A) Schematic of microbe-mediated energy fluxes in the Thermodynamic Microbial Consumer Resource Model. Each cell of species *i*(= 1, 2, … *S*) supplies itself with energy through import of resources, generating an incoming energy flux $J_{i\alpha }^{\text{in}}$ for each resource type *α*(= 1, 2, … *M*). A fraction *l*_*α*_ of this energy leaks back into the environment in the form of metabolic byproducts, with each byproduct type carrying an outgoing energy flux $J_{i\beta }^{\text{out}}=\sum_{\alpha }\;l_{\alpha }D_{\beta \alpha }J_{i\alpha }^{\text{in}}$. The remaining energy, $J_{i}^{\text{grow}}$, is used to replicate the cell. (B) Each species is defined by a vector of consumer preferences that encode its capacity for harvesting energy from each resource type. These vectors comprise a consumer matrix *c*_*iα*_. (C) A regional pool of species is randomly generated, and communities are initialized with random subsets of these species to simulate stochastic colonization. (D) Each community is supplied with a constant flux *κ*_0_ of a single resource type (*α* = 0), and all resources are continuously diluted at a fixed rate $\tau_{R}^{-1}$. (E) Consumer populations *N*_*i*_ and resource concentrations *R*_*α*_ as a function of time for two realizations of this model, with low (*l* = 0.001) and high (*l* = 0.8) levels of uniform metabolic leakage (see S1 Text Section 2B for parameters).

The starting point for our analysis is a new model that adapts MacArthur’s Consumer Resource Model [7] to the microbial context by including energetics, stochastic colonization, and the exchange and consumption of metabolites. We consider the population dynamics of *S* species of consumers (e.g., microbes) competing for *M* types of substitutable resources. We are interested in large, diverse ecosystems where *S*, *M* ≫ 1. A schematic summarizing our model is shown in Fig 1.

A natural setting for considering substitutable resources is when all essential biomass components are supplied in excess, and the limiting factor for growth is the supply of usable energy. In this context, one only needs to keep track of resources from which energy can be harvested. All other nutrients are included implicitly, under the assumption that some of the energy budget is used to import whatever materials are required for growth and reproduction. Terminal waste products from which no more energy can be extracted are likewise treated implicitly, and are not included among the *M* resource types.

In our model, the rate at which an individual of species *i* harvests energy from resource *α* depends on the resource concentration *R*_*α*_ as well as on the consumer’s vector of resource preferences *c*_*iα*_ through the expression:

$$\begin{array}{c} J_{i\alpha }^{\text{in}}=w_{\alpha }\sigma \left(c_{i\alpha }R_{\alpha }\right), \end{array}$$ (1)

where *σ*(*x*) encodes the functional response and has units of mass/time, while *w*_*α*_ is the energy density of resource *α* with units of energy/mass. In the microbial context the consumer preferences *c*_*iα*_ can be interpreted as expression levels of transporters for each of the resources. In the main text, we focus on Type-I responses where *σ*(*x*) = *x*, and we set *w*_*α*_ = 1 for all *α*, but most of our results still hold when *σ*(*x*) is a Monod function or the *w*_*α*_ are randomly sampled, as shown in Section 3 of S1 Text.

We model leakage and secretion by letting a fraction *l*_*α*_ of this imported energy return to the environment, so that the power available to the cell for sustaining growth is equal to

$$\begin{array}{c} J_{i}^{\text{grow}}=\sum_{\alpha }\left(1-l_{\alpha }\right)J_{i\alpha }^{\text{in}}. \end{array}$$ (2)

This parameterization guarantees that the community does not spontaneously generate usable energy in violation of the Second Law of Thermodynamics. We assume that a fixed quantity *m*_*i*_ of power per cell is required for maintenance of a steady population of species *i*, and that the per-capita growth rate is proportional to the remaining energy flux, with proportionality constant *g*_*i*_. In typical experimental conditions, cell death is negligible, and *m*_*i*_ is the energy harvest required for the replication rate to keep up with the dilution rate. Under these assumptions, the time-evolution of the population size *N*_*i*_ of species *i* can be modeled using the equation

$$\begin{array}{c} \frac{dN_{i}}{dt}=g_{i}N_{i}[J_{i}^{\text{grow}}-m_{i}]. \end{array}$$ (3)

The leaked energy flux $J_{i}^{\text{out}}=\sum_{\alpha }\;l_{\alpha }J_{i\alpha }^{\text{in}}$ from each cell of species *i* is partitioned among the *M* possible resource types via the biochemical pathways operating within the cell. We assume that all species share a similar core metabolism, encoded in a matrix *D*_*βα*_. Each element of *D*_*βα*_ specifies the fraction of leaked energy from resource *α* that is released in the form of resource *β* (note that by definition, ∑_*β*_ *D*_*βα*_ = 1). Thus, in our model the resources that are excreted into the environment are intimately coupled to the resources a cell is consuming. The outgoing energy flux contained in metabolite *β* is given by

$$\begin{array}{c} J_{i\beta }^{\text{out}}=w_{\beta }\nu_{i\beta }^{\text{out}}=\sum_{\alpha }D_{\beta \alpha }l_{\alpha }J_{i\alpha }^{\text{in}}. \end{array}$$ (4)

The dynamics of the resource concentrations depend on the incoming and outgoing mass fluxes $\nu_{i\alpha }^{\text{in}}=\sigma \left(c_{i\alpha }R_{\alpha }\right)$ and $\nu_{i\alpha }^{\text{out}}$, which are related to the energy fluxes via the energy densities *w*_*α*_. In terms of these quantities, we have

$$\begin{array}{c} \frac{dR_{\alpha }}{dt}=h_{\alpha }+\sum_{j}N_{j}\left(\nu_{j\alpha }^{\text{out}}-\nu_{j\alpha }^{\text{in}}\right), \end{array}$$ (5)

with *h*_*α*_ encoding the dynamics of externally supplied resources. In this manuscript, we focus on the case where the microbial communities are grown in a chemostat with a single externally supplied resource *α* = 0 (Fig 1). In this case, the resource dynamics can be described by choosing $h_{\alpha }=\kappa_{\alpha }-\tau_{R}^{-1}R_{\alpha }$, with all the *κ*_*α*_ set to zero except for *κ*_0_. These equations for *N*_*i*_ and *R*_*α*_, along with the expressions for $J_{i\alpha }^{\text{in}}$ and $J_{i\alpha }^{\text{out}}$, completely specify the ecological dynamics of the model.

This model has been implemented in a freely available open-source Python package “Community Simulator.” The package can be downloaded from https://github.com/Emergent-Behaviors-in-Biology/community-simulator.

## Results

To assess the typical community structure and resource pool stability for ecosystems obeying Eqs (1)–(5), we randomly generated an *M* × *M* metabolic matrix *D*_*αβ*_, and a binary *S* × *M* consumer preference matrix *c*_*iα*_ with *S* = 200 species and *M* = 100 resources. We chose *c*_*iα*_ so that each species had 10 preferred resource types on average, with *c*_*iα*_ = 1, while the rest of the resources were consumed at a baseline level of *c*_*iα*_ = 0.01. The metabolic matrix *D*_*αβ*_ was sampled from a Dirichlet distribution, which guarantees that all the columns sum to 1 as required by the definition of this parameter. In Section 3 of S1 Text, we show that the qualitative patterns we observe are unchanged if *c*_*iα*_ is drawn from a Gaussian or Gamma distribution, or if the *D*_*αβ*_ matrix is made less sparse. The full sampling procedure is detailed in S1 Text Section 1.

We chose our units of energy flux such that the mean maintenance cost *m*_*i*_ over all species in the regional pool is equal to 1. To break ties between species with similar consumption profiles, we added a Gaussian random offset to the *m*_*i*_ of each species with standard deviation 0.1. In S12 Fig., we show that these intrinsic fitness differences do not dominate the ecological dynamics, and that many species with relatively high maintenance costs are able to reach large population sizes in the steady-state communities. Finally, we set all the *w*_*α*_ equal to 1, and made all the leakage fractions identical, with *l*_*α*_ = *l* for all *α*.

To assess the amount of variability in the results, we initialized 10 different communities by seeding each one with a random subset of 100 species from the full 200-species pool. This simulates the stochastic colonization frequently observed in microbial ecosystems, where the community composition can randomly vary depending on the set of microbes this particular local environment happened to be exposed to [37]. Fig 1 shows typical dynamical trajectories in the presence of high (*l* = 0.8) and extremely low leakage (*l* = 0.001).

### Available energy fluxes drive a transition between a “resource-limited” and “diverse” regime

**Fig 2:**
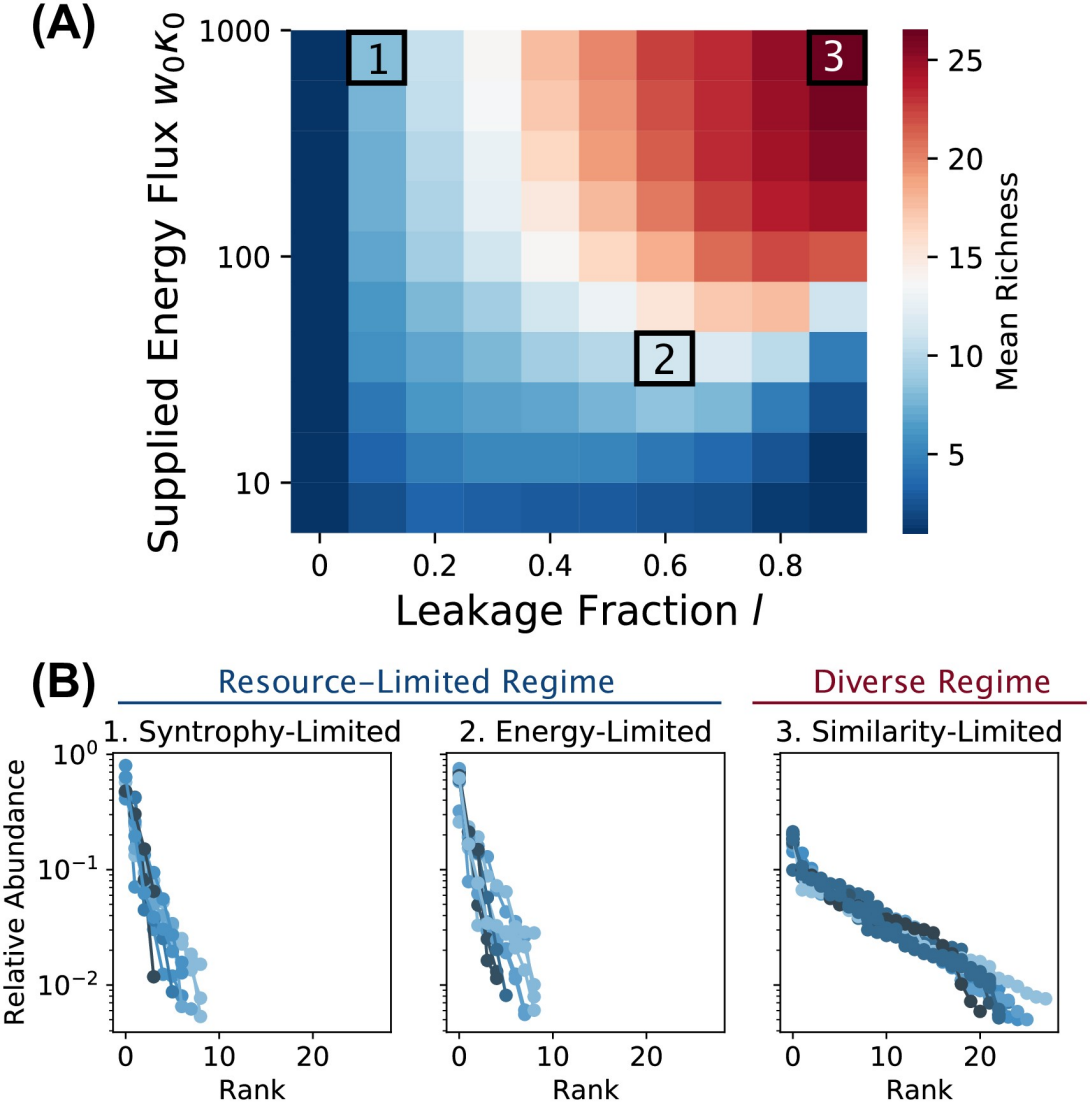
Steady-state richness as a function of metabolic leakage *l* and externally supplied energy flux *w*_0_*κ*_0_. We generated 200 species, initialized 10 communities of 100 species each from this pool, and ran the dynamics to steady state under different combinations of *w*_0_*κ*_0_ and *l* (see main text and S1 Text Section 2B for parameters). (A) Heat map summarizing all simulations, colored by the average number of surviving species per steady-state community (“Richness”). Slices through the heat map are plotted in S3 Fig. (B) Community compositions are displayed as rank-abundance curves for three illustrative *w*_0_*κ*_0_, *l* combinations (colored by community richness): (1) “syntrophy-limited” (*w*_0_*κ*_0_ = 1000, *l* = 0.1), (2) “energy-limited” (*w*_0_*κ*_0_ = 28, *l* = 0.6) and (3) “similarity-limited” (*w*_0_*κ*_0_ = 1000, *l* = 0.9). The lines are assigned different shades for clarity. The first two examples are parts of the same resource-limited regime, manifesting similar statistical properties. The plots are truncated at a relative abundance of 0.5%; see S4 Fig. for full data.

Our numerical simulations display a transition between two qualitatively different community structures as we vary the externally supplied energy flux *w*_0_*κ*_0_ and the leakage/syntrophy *l*. In the “thermodynamic limit” of *M*, *S* → ∞, the communities exhibit signatures of a phase transition analogous to those found in disordered systems in physics (see Discussion and S1 Text Section 5). Fig 2 shows the effect of this transition on community diversity at our chosen finite values of *S* and *M*. At low levels of energy flux or syntrophy, the diversity is severely limited by resource availability. In the limit of high supplied energy flux and high leakage, a maximally diverse regime is obtained, where the number of surviving species is limited only by the similarity between consumption profiles within the regional species pool, in accordance with classical niche-packing theory [7] as we will discuss below.

### The resource-limited and diverse regimes produce different patterns of energy flux

**Fig 3:**
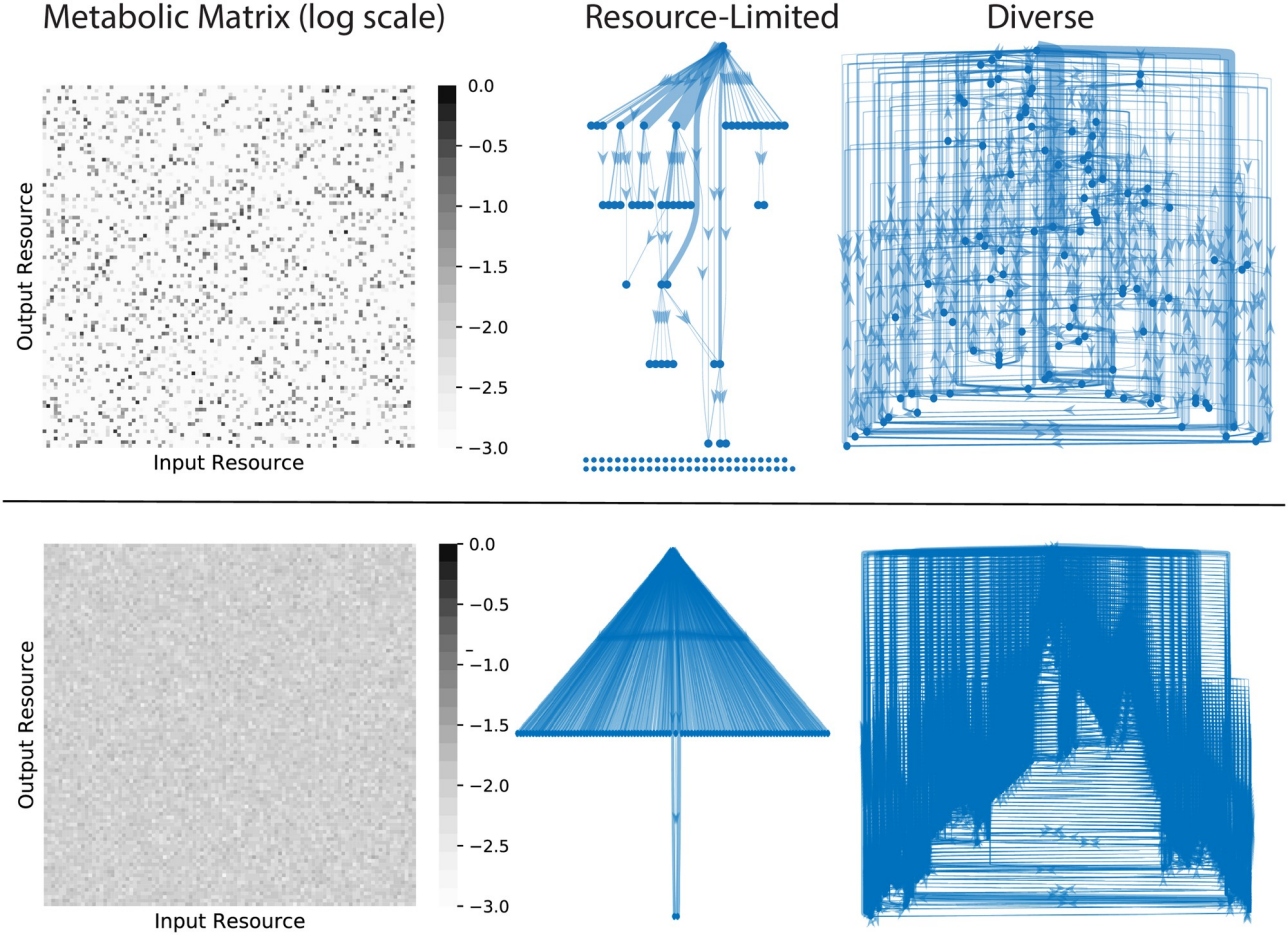
Energy flux networks differ in the two regimes. Community-scale energy flux networks are plotted for a characteristic example from the diverse and resource limited regimes and two different choices of metabolic matrix *D*_*αβ*_. The color of each pixel in the heat maps indicates the logarithm (base 10) of the corresponding matrix entry. In the networks, each node represents one of the *M* = 100 resource types. Edges represent steady-state energy flux from one resource type into another, mediated by consumer metabolism and leakage/secretion. The thickness of each edge is proportional to the flux magnitude, and edges with magnitudes less than 1% of the maximum flux are not displayed. The single node at the top of each graph is the externally supplied resource, and the rows of nodes at the bottom are resources that are not connected to the external supply by any flux above the 1% threshold. A topological analysis of the flux networks of all the simulated communities can be found in S10 Fig.

The difference between the two regimes is most apparent from the perspective of the energy flux networks. Because our model explicitly accounts for the flow of energy from one resource type into another, we can compute all the steady-state fluxes and represent them graphically, as shown in Fig 3 for some representative examples. Each node in this network is a resource type, and each directed edge represents the steady-state flux *J*_*βα*_ of energy conversion from resource *α* to resource *β*, mediated by one or more syntrophic consumers:

$$\begin{array}{c} J_{\beta \alpha }=\sum_{i}N_{i}J_{i\beta \alpha }^{\text{out}}=D_{\beta \alpha }l_{\alpha }\sum_{i}N_{i}w_{\alpha }c_{i\alpha }R_{\alpha }. \end{array}$$ (6)

The resource-limited regime produces a unidirectional flow of energy, which is converted from the externally supplied resource type into an orderly succession of secreted resources. For the sparse metabolic matrix shown in the top row of Fig 3, most resource types also have extremely small incoming flux vectors in this regime, with magnitudes less than 1% the size of the largest flux in the network. The diverse regime displays a qualitatively different structure, where all resources have significant incoming fluxes (regardless of the choice of *D*_*αβ*_), and the large number of loops in the network makes it impossible to put the resource types into any definite order. In S10 Fig., we plot the fraction of samples from Fig 2 whose (pruned) flux networks are free of cycles, and confirm that this observation is generic. The dramatic contrast between the community-level metabolism of the two regimes affects many other global features of the ecosystem, which we will explore in the following sections.

### The two regimes have distinct functional structures

**Fig 4:**
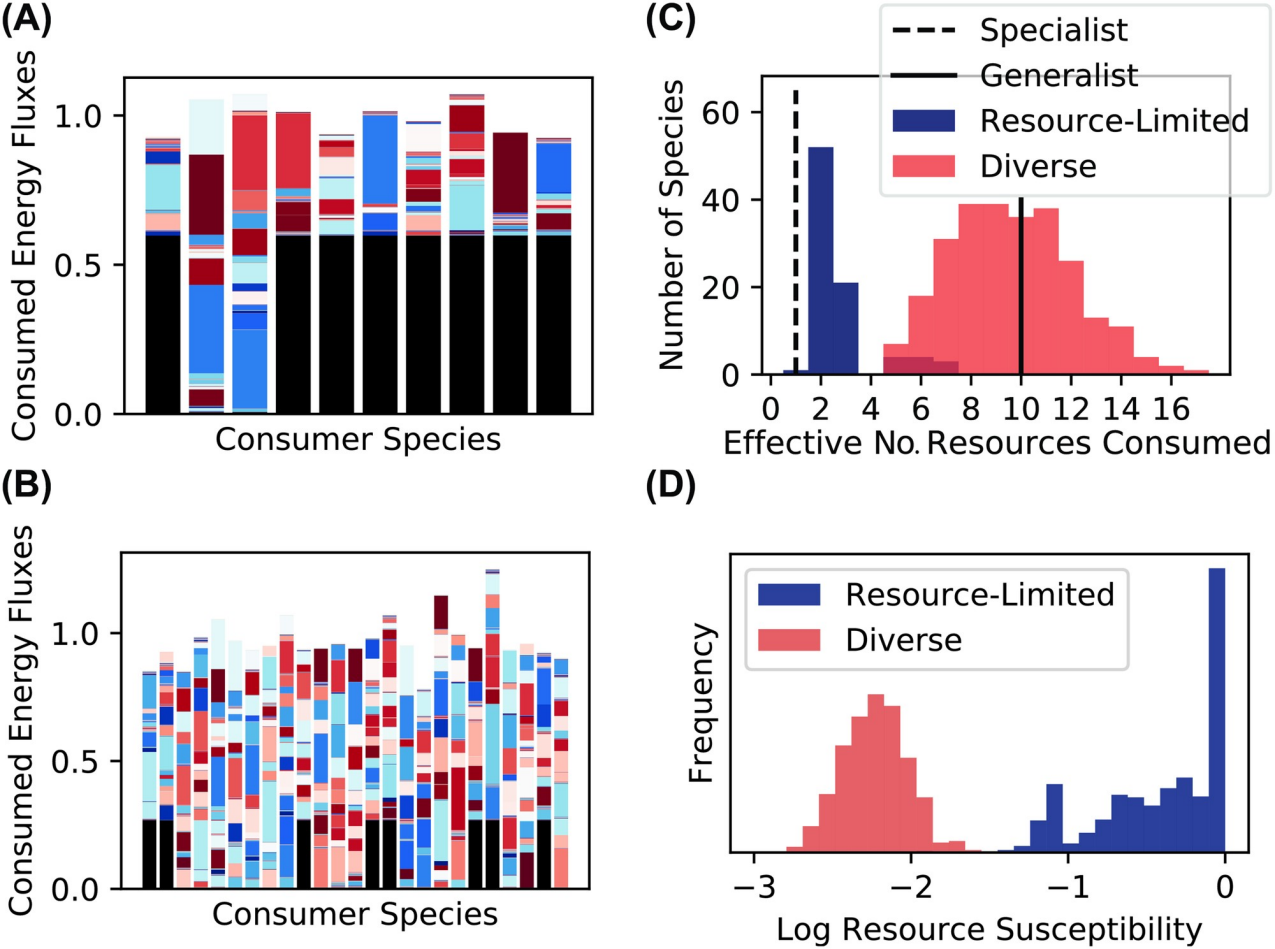
Structure and stability of resource dynamics depend on ecological regime. (A) Consumed energy fluxes $\left(1-l\right)J_{i\alpha }^{\text{in}}$ for each of the ten surviving species in a resource-limited community (example 2 from Fig 2). The black portion of the bar is the flux $\left(1-l\right)J_{i0}^{\text{in}}$ due to the externally supplied resource, and the colored bars represent the contributions of the other resources. Since these communities have reached the steady state, Eq (3) implies that the total height of each bar equals the maintenance cost *m*_*i*_ of the corresponding consumer species. (B) Same as previous panel, but for a community from the diverse regime (example 3 from Fig 2). (C) Simpson diversity $M_{i}^{\text{eff}}$ of steady-state flux vector $J_{i\alpha }^{\text{in}}$ for each species from examples 2 (resource-limited) and 3 (diverse) in Fig 2. Vertical lines indicate the values of this metric when all the flux is concentrated on a single resource (“Specialist”), and where it is evenly spread over ten resource types (“Generalist”). (D) Logarithm of susceptibility $\log_{10}\partial {\bar{R}}_{\alpha }/\partial \kappa_{\alpha }$ of community-supplied resources (*α* ≠ 0) to addition of an externally supplied flux *κ*_*α*_ in these two examples.

To better understand the behavior of consumers in the two regimes, we examined the functional traits of members of typical communities in each one. In the resource-limited regime, many surviving species derive most of their energy directly from the externally supplied resource (Fig 4A). In the diverse regime, by contrast, only a minority of the steady-state community members can consume this resource at all, and even these species receive most of their energy from a diverse array of metabolic byproducts (Fig 4B). We quantified this observation using the Simpson Diversity $M_{i}^{\text{eff}}$ of the incoming resource flux vectors $J_{i\alpha }^{\text{in}}$, which measures the effective number of resources consumed by each species, and is closely related to the inverse participation ratio in statistical physics. The Simpson Diversity is defined by

$$\begin{array}{c} M_{i}^{\text{eff}}={\left[\sum_{\alpha }{\left(\frac{J_{i\alpha }^{\text{in}}}{J_{i}^{\text{in}}}\right)}^{2}\right]}^{-1}, \end{array}$$ (7)

where $J_{i}^{\text{in}}=\sum_{\alpha }\;J_{i\alpha }^{\text{in}}$ is the total incoming energy flux for each cell of this species. $M_{i}^{\text{eff}}$ approaches 1 for species that obtain the bulk of their energy from a single resource type and approaches *M* when all resource types are consumed equally. In the resource-limited regime, the distribution of these values is sharply peaked around 2. In the diverse regime, the peak is located around 10, which is the average number of resources with high transporter expression in our binary sampling scheme for *c*_*iα*_. This shows that most community members in the diverse regime utilize multiple energy sources, with the incoming flux spread evenly over all resource types they are capable of consuming.

### Responses to resource perturbations differ in the two regimes

Another important property of microbial ecosystems is how they respond to environmental perturbations. Previous theoretical studies have shown that sufficiently diverse communities can “pin” the resource concentrations in their local environment to fixed values, which are independent of the magnitude of externally supplied fluxes [21, 38, 39]. In these studies, resource pinning occurs only when the community saturates the diversity bound imposed by the principal of competitive exclusion, i.e. when the number of coexisting species is at least as large as the number of resource types. Such maximally diverse communities typically require fine-tuning of the resource utilization profiles or imposition of universal efficiency tradeoffs in cellular metabolism.

In our stochastically assembled communities, the diversity is always much lower than the number of resource types, so we hypothesized that the resource concentrations should not be pinned. To test this idea, we measured the response of the steady-state concentrations ${\bar{R}}_{\alpha }$ to changes in external supply rates *κ*_*α*_, in terms of the “resource susceptibilities” $\partial {\bar{R}}_{\alpha }/\partial \kappa_{\alpha }$ plotted in Fig 4D [34]. Our hypothesis was valid in the resource-limited regime, where many resource susceptibilities are comparable to the susceptibility in the empty chemostat $\partial {\bar{R}}_{\alpha }/\partial \kappa_{\alpha }=\tau_{R}=1$. But in the diverse regime, we were surprised to find that the susceptibilities are 100 times smaller than this maximum value. This suggests that resource pinning may be a generic phenomenon, observable in real ecosystems when the energy supply is sufficiently large.

### Niche overlap limits richness in diverse regime

In the diverse regime, the number of coexisting species (“richness”) is not limited by energy availability or by access to secreted metabolites, but is still much less than the maximal value of *M* = 100 set by the competitive exclusion principle [8], even though almost all *M* resource types are present at non-negligible levels (as shown in S11 Fig.). We hypothesized that the diversity in this regime is limited by the degree of similarity between consumption preferences of members of the regional species pool. This can be quantified in terms of the niche overlap [9, 40], whose average value in a large regional pool is given by:

$$\begin{array}{c} \langle \rho_{ij}\rangle \equiv \langle \frac{\sum_{\alpha }\;c_{i\alpha }c_{j\alpha }}{\sqrt{\sum_{\alpha }\;c_{i\alpha }^{2}\sum_{\alpha }\;c_{j\alpha }^{2}}}\rangle =\frac{{\langle c_{i\alpha }\rangle }^{2}}{\langle c_{i\alpha }^{2}\rangle }. \end{array}$$ (8)

**Fig 5:**
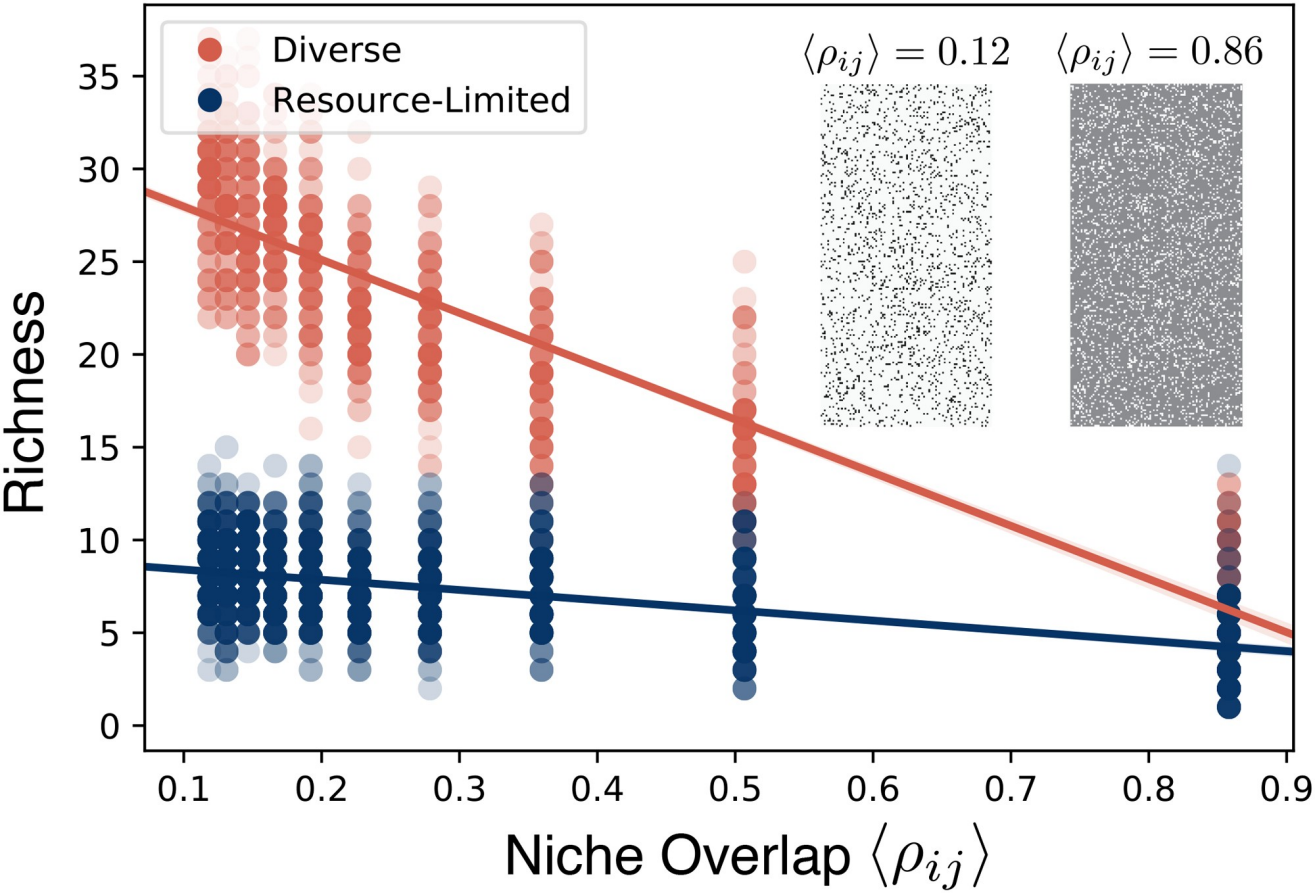
Richness of diverse regime depends on generalized niche-overlap. We took the values of supplied energy flux *w*_0_*κ*_0_ and leakage fraction *l* from the three examples highlighted in Fig 2, and varied the average niche overlap 〈*ρ*_*ij*_〉 between members of the metacommunity. For each *w*_0_*κ*_0_, *l* combination and each value of 〈*ρ*_*ij*_〉, we generated 10 pools of 200 species, initialized 10 communities of 100 species each from this pool, and ran the dynamics to steady state. The steady-state richness of each community is plotted against the niche overlap. Points are colored by their regime (diverse or resource-limited), and solid lines are linear regressions. Inset: *c*_*iα*_ matrices that define the regional pool for two different levels of overlap, with dark squares representing high consumption coefficients.

Fig 5 shows how the richness varies as a function of 〈*ρ*_*ij*_〉. In the diverse regime the mean richness decreases approximately linearly with increasing overlap. The richness of the resource-limited regime, on the other hand, has only a very weak dependence on the niche overlap. These results suggest that the distribution of consumption preferences in the regional pool is the primary driver of community assembly in the diverse regime. Importantly, non-zero niche overlap limits the number of coexisting species well below the upper bound imposed by the competitive exclusion principle.

### Nestedness and other large-scale beta-diversity patterns

**Fig 6:**
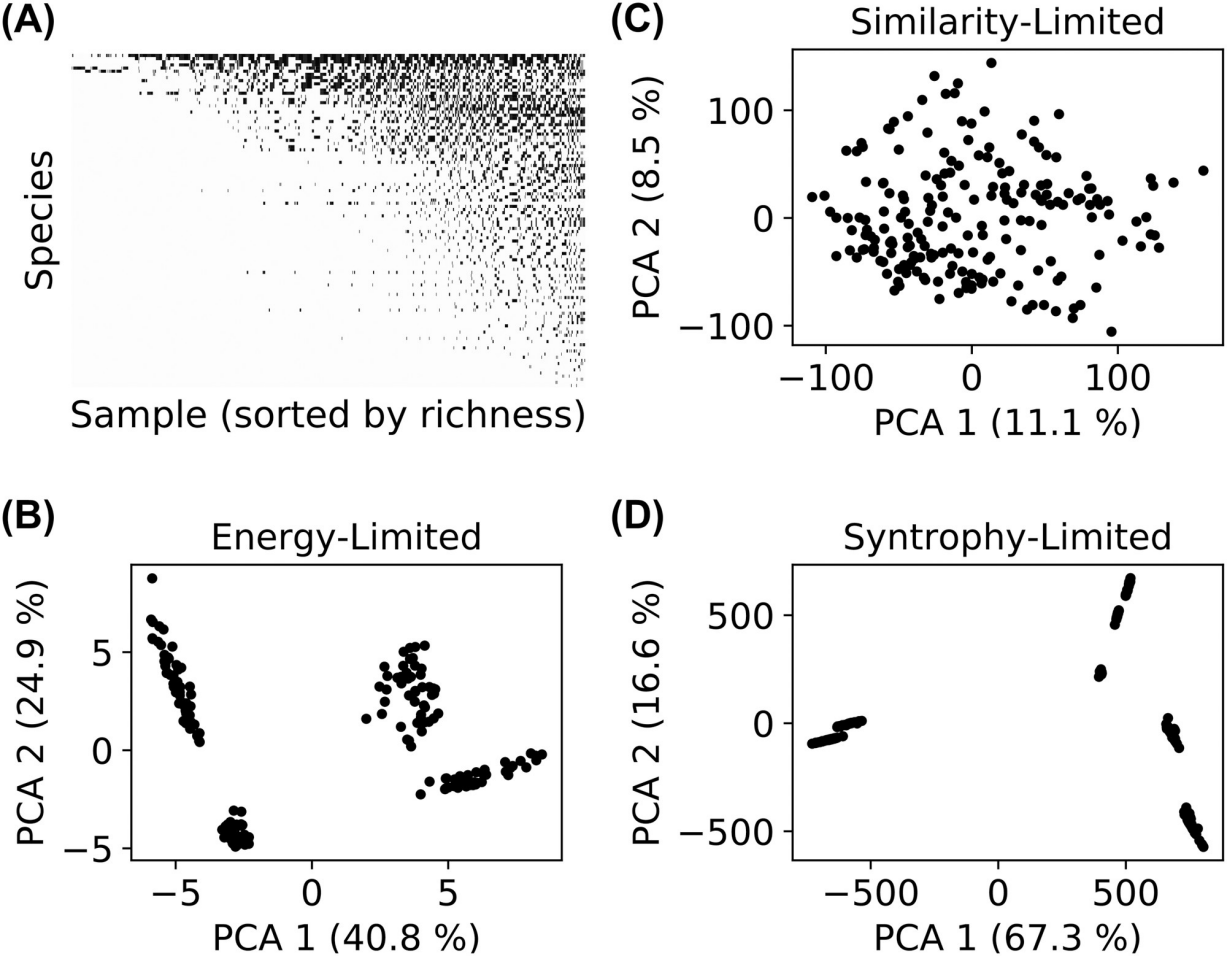
Resource-limited regime features community-level environmental filtering. (A) Presence (black) or absence (white) of all species in all 1,000 communities from the original simulations of Fig 2. (B, C, D) We initialized 200 new communities for each of the three examples highlighted in Fig 2A, by randomly choosing sets of 100 species from the regional pool. Each panel shows the projection of final community compositions {*N*_*i*_} onto the first two principal components of the set of compositions.

Our aim in developing this model is to identify and understand generic patterns in community structure, that are independent of particular biological details. In large-scale surveys of natural communities, subject to many sources of noise and environmental heterogeneity, one expects that only sufficiently generic patterns will be detectable. The simplest observable to examine in such survey data is the list of species that are present or absent in each sample. We obtained these presence/absence vectors from the simulations of Fig 2, and found that when we sorted species by prevalence (rows in Fig 6A) and samples by richness (columns in Fig 6A), the community composition generically exhibited a nested structure—less diverse communities tended to be subsets of more diverse communities [41, 42]. We quantified this result using an established nestedness metric, as described in S1 Text and S7 Fig., and found that the actual nestedness exceeds the mean value for a randomized null model by more than 100 standard deviations. This suggests that nested structures may generically emerge in community assembly through the interplay of stochastic colonization, competition, and environmental filtering.

Next, we asked if we large-scale beta-diversity patterns could be used to distinguish the resource-limited and diverse regimes. We initialized 200 new communities with 100 randomly chosen members from the full regional species pool and simulated these communities to steady state in both the resource-limited and diverse regimes (see S1 Text Section 2B for details). This sub-sampling of the full regional species pools mimics the effect of stochastic colonization, where a different random subset of species seeds each community. To better understand beta-diversity signatures in the two regimes, we performed a Principal Component Analysis (PCA) on community composition and projected the data onto the first two principal components, as shown in Fig 6B–6D. In the resource-limited regime, the communities form distinct clusters that are distinguished by different highly abundant species. This suggests that harsh environments only allow a few species from the regional pool to rise to dominance, and that these dominant species induce clustering of communities. Such “enterotype”-like behavior is a common feature observed in many microbial settings [43]. In contrast, the diverse regime exhibited neither well-defined clusters nor dominant, highly abundant species.

### Comparison to microbial datasets

**Fig 7:**
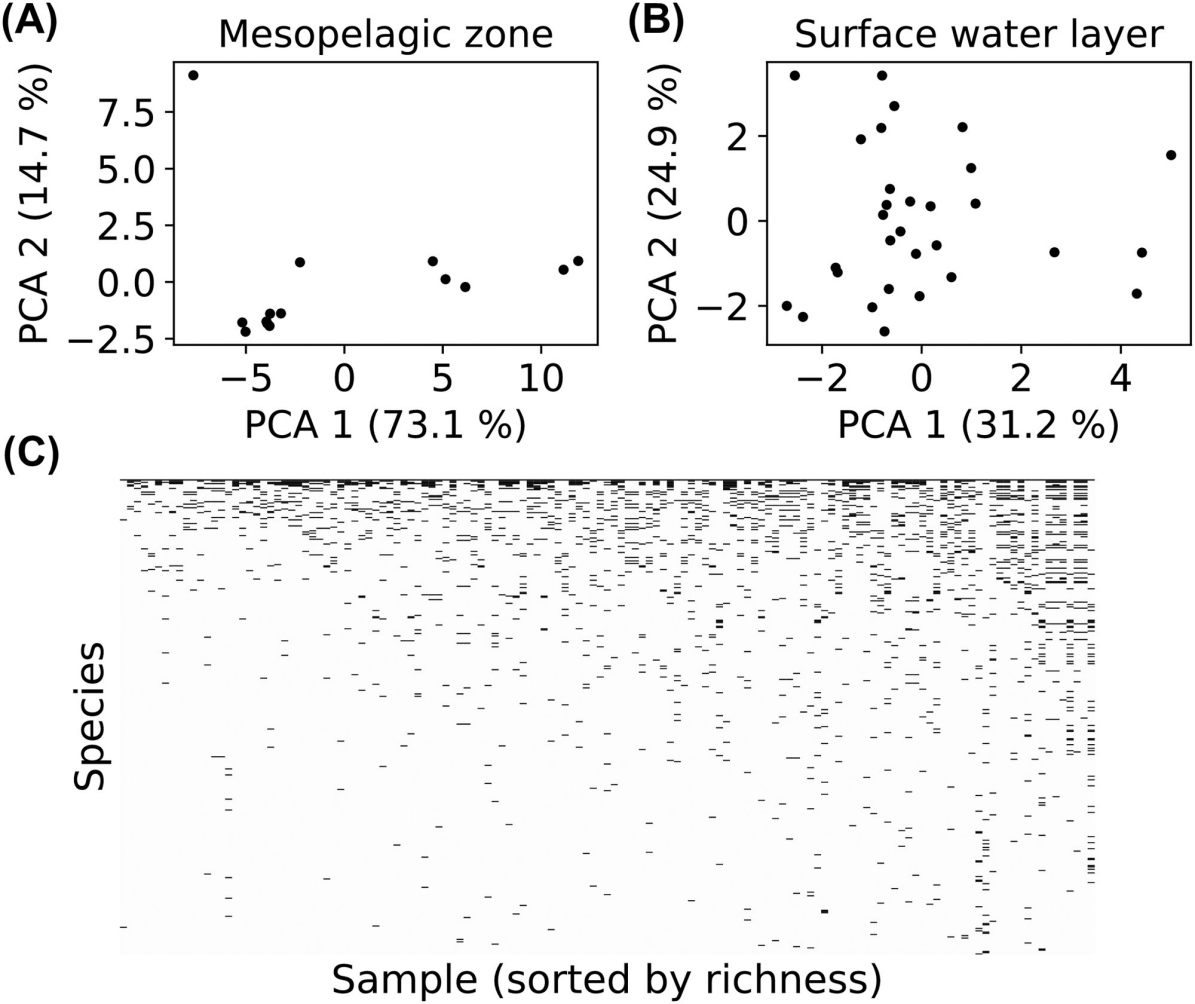
Ecological regimes and nestedness in microbiome data. (A) 16S OTU compositions of tropical mesopelagic zone samples from the Tara Oceans database, collected at a depth of 200 to 1,000 meters [36]. Each dot is the projection of one sample onto the first two principal components of the collection of mesopelagic zone samples. (B) Same as A, but for tropical surface water layer samples, collected at a depth of 5 meters. (C) Presence (black) or absence (white) of each OTU above 0.5% relative abundance across all Tara Oceans samples.

The preceding results suggest that the resource-limited and diverse regimes can be distinguished using beta-diversity patterns. Rigorous testing of this prediction is beyond the scope of the present work. But as an illustration of the kind of data we hope to explain, we examined the natural gradient of solar energy supply in the Tara Oceans survey, which collected microbial community samples from a range of depths across the world’s oceans [36]. Explicitly including light as an energy source would require some modification to the structure of the model equations, but we expect that the large-scale features of sufficiently diverse ecosystems should not be sensitive to changes involving just one resource. We analyzed the 16S OTU composition of tropical ocean communities for all 30 sea-surface samples, where solar energy is plentiful, and all 13 samples from the deep-sea mesopelagic zone where energy fluxes are limited. We projected these composition vectors onto their first two principal components as in Fig 6 above, and plot the results in Fig 7. The sea surface data superficially resembles our diverse regime, with a relatively uniform distribution of possible community compositions. In contrast, the Mesopelagic Zone is similar to our resource-limited regime: the dominance of the most abundant species is much more pronounced, and the compositions appear to cluster into four discrete types. While these results are consistent with our model predictions, the number of samples at each depth is still too small to draw any definitive conclusions about clustering.

As mentioned above, our model also gives a natural explanation for the nestedness in the Earth Microbiome Project community composition data [1], suggesting that it may be a natural byproduct of complex microbial communities shaped by competition, environmental heterogeneity, and stochastic colonization. To test how generic this feature is, we plotted presence/absence community compositions of all samples from the Tara Oceans dataset, sorting samples by richness and OTU’s (“species”) by prevalence. Each sample contains thousands of low-abundance OTU’s, which can obscure ecological patterns through their susceptibility to sequencing noise and transient immigration. We therefore imposed a 0.5% relative abundance threshold for an OTU to count as “present.” The resulting pattern in Fig 7 is qualitatively similar to our simulations (Fig 6D), and to the phylum-level data of the Earth Microbiome Project [1], with the region below the diagonal significantly less populated than the region above the diagonal (although the signal is much weaker). In S1 Text Section 4 and S7 Fig., we quantify the nestedness using the same metric employed in the Earth Microbiome Project analysis [1, 44], and show that the score is significantly higher than the mean scores from two standard null models.

## Discussion

Advances in sequencing technology have opened new horizons for the study of microbial ecology, generating massive amounts of data on the composition of both natural and synthetic communities. But the complexity of these systems make it difficult to extract robust general principles suitable for guiding medical and industrial applications. Numerical simulations provide a powerful tool for addressing this problem. By rapidly iterating numerical experiments under a variety of modeling choices with random parameters, one can identify robust patterns and use the resulting insights to guide targeted experiments.

Following this strategy, we developed a thermodynamic consumer resource model that explicitly includes energetic fluxes and metabolically mediated cross-feeding and competition. Using this model, we identified two qualitatively distinct regimes as we varied the amount of energy supplied to ecosystem and the fraction of energy leaked back into the environment: a low diversity “resource-limited” regime and a “diverse” regime. The structure of the resource-limited regime is strongly constrained by species- and community-level environmental filtering. Each community is dominated by a handful of species, making the community properties sensitive to the idiosyncratic characteristics of these species and susceptible to environmental fluctuations. In the diverse regime, communities exhibit more universal features because they substantially engineer their environments. In particular, the concentrations of resources at steady state are more narrowly distributed and insensitive to perturbations in the external supply rates. Moreover, each species draws its energy roughly equally from all resources, rather than subsisting on the externally supplied resource as in the resource-limited regime.

The emergence of environmental engineering from this community-scale model makes it a valuable tool for testing and refining existing conceptual frameworks employed by empirical biologists [45]. A major limitation of the dominant paradigms for evolution and ecology from the last century is the implicit assumption of a constant environment [46]. The generalized Lotka-Volterra model, for example, remains a standard lens for reasoning about ecological dynamics, both quantitatively and qualitatively [47–49]. It assumes that the dynamics emerge from the sum of pairwise interactions among species, and that the sign and strength of these interactions are intrinsic properties of the species. This can be a good assumption in some circumstances [47, 48], but fails to accurately describe the behavior of simple models that explicitly account for the state of the environment [50]. Our work provides a starting point for determining the conditions under which pairwise models will generically succeed or fail in describing the behavior of large ecosystems.

Our model complements other recent efforts at understanding microbial community ecology. Taillefumier *et al*. proposed a similar model of metabolite exchange, and focused on the case where the number of resource types *M* is equal to 3 [21]. In this case, repeated invasion attempts from a large regional species pool produced optimal combinations of metabolic strategies. Goyal *et al*. examined the opposite limit, with *M* = 5, 000, but allowed each species to consume only one type of resource [22]. This generated communities with a tree-like metabolic structure, where each species depends directly on another species to generate its unique food source. In our model, the large number of resource types (*M* = 100 in the current study) makes spontaneous strategy optimization extremely unlikely. And our generic protocol for sampling the metabolic matrix *D*_*αβ*_ allows a variety of community-level energy flux topologies to emerge, as illustrated in Fig 3, which can sometimes be quite different from the tree networks of Goyal *et al*. The absence of highly specialized metabolic structure in our model makes it especially well-suited for interpreting patterns in large-scale sequence-based datasets such as the Earth Microbiome Project [1].

Our model predictions can also be directly tested using experiments with natural communities in synthetic laboratory environments [17, 51]. Our model predicts that beta-diversity patterns and community-level metabolic networks can be significantly altered by increasing the ecosystem’s energy supply, inducing a transition from the resource-limited to the diverse regime. In the experimental set-up of [51], this can be done by directly adding chitinase enzymes to the sludge reactor to increase the degradation of chitin-based organic particles on which the ocean-derived microbial communities subsist. One could then look for shifts in the resulting diversity patterns, and observe any changes in the topology of the metabolic flux network using isotope labeling.

In this work we have largely confined ourselves to studying steady-state properties of well-mixed microbial communities. Microbial communities often exhibit complex temporal dynamics with well-defined successions [51–53]. It will be interesting to explore these dynamical phenomena using our model. It is also well established that spatial structure can give rise to new ecological phenomena [54, 55] and an important area of future work will be to better explore the role of space in microbial community assembly.

Finally, we have obtained strong numerical evidence that the two regimes are separated by a phase transition, which is likely closely related to disorder-induced phase transitions in statistical physics [32]. In Supporting Text Section 5, we examine the steady-state richness in the three examples of Fig 2 under increasing values of *M* from *M* = 40 to *M* = 560. We find that the richness is proportional to *M* in the diverse regime, but scales sub-linearly with *M* in both examples from the resource-limited regime. In the *M* → ∞ limit, therefore, we expect to find a sharp line between the regimes, with the ratio of the richness to *M* vanishing on the resource-limited side. But we do not yet know the exact location of this boundary, or the critical exponents describing the behavior of the system near the transition.

## Supporting information

S1 Text Simulation details. (PDF)

S1 Fig Richness vs. *w*_0_*κ*_0_ and 〈*l*_*α*_〉 under different modeling choices. (PDF)

S2 Fig Simpson Diversity vs. *w*_0_*κ*_0_ and 〈*l*_*α*_〉 under different modeling choices. (PDF)

S3 Fig Richness (blue solid) and Simpson Diversity (red dotted) for cuts through the heat map. (PDF)

S4 Fig Rank-abundance curves for three representative examples. (PDF)

S5 Fig Effective number of resources consumed under different modeling choices. (PDF)

S6 Fig Resource susceptibility. (PDF)

S7 Fig Quantification of nestedness. (PDF)

S8 Fig Scaling of consumer richness with system size. (PDF)

S9 Fig Scaling of other observables. (PDF)

S10 Fig Flux network topology. (PDF)

S11 Fig Number of available resources. (PDF)

S12 Fig Maintenance costs *m*_*i*_. (PDF)
