# Supplementary material for: Available energy fluxes drive a transition in the diversity, stability, and functional structure of microbial communities
Source: PLoS Comput Biol. 2019 Feb 5;15(2):e1006793. doi: 10.1371/journal.pcbi.1006793 (PMC6386421; doi:10.1371/journal.pcbi.1006793)
Supplement: S1 Text — (PDF) [file pcbi.1006793.s001.pdf]

Supplementary Information for

Available energy fluxes drive a transition in the diversity, stability, and functional structure of microbial communities

Robert Marsland III, Wenping Cui, Joshua Goldford, Alvaro Sanchez, Kirill Korolev and Pankaj Mehta

Corresponding Author: Robert Marsland III.
E-mail: marsland@bu.edu

Contents

1 Model details **2**
A Generalities . . . 2
B Input fluxes and output partitioning . . . 2
C Choosing consumer preferences . . . 3
D Constructing the metabolic matrix . . . 4

2 Simulation and data analysis **4**
A The Community Simulator . . . 4
B Simulation Details . . . 5
C Susceptibilities . . . 5
D Niche Overlap . . . 6
E Beta Diversity . . . 6
F Data Format . . . 6

3 Robustness of qualitative results **6**
A Type-II Growth . . . 7
B Metabolic Matrices . . . 7
C Randomness in w_α and l_α . . . 7
D Gaussian and Gamma Sampling . . . 7

4 Quantification of Nestedness **7**

5 Numerical Evidence of a Phase Transition **8**

In this document, we provide a full explanation of the model employed in the article "Available energy fluxes drive a transition in the diversity, stability, and functional structure of microbial communities." We also describe its numerical implementation, and present additional data illustrating the robustness of the qualitative results. Finally, we present preliminary evidence for the existence of a bona fide phase transition in the $M \to \infty$ limit. All data and code for generating figures can be found at https://github.com/Emergent-Behaviors-in-Biology/, as described in Section 2 below.

1. Model details

A. Generalities. We begin by defining an *energy flux* into a cell $J^{\rm in}$, an energy flux that is used for growth $J^{\rm growth}$, and an outgoing energy flux due to byproduct secretion $J^{\rm out}$. Energy conservation requires

$$J^{\rm in} = J^{\rm growth} + J^{\rm out} \qquad [1]$$

for any reasonable metabolic model. Now consider a model with M resources R_β with $\beta = 1 \dots M$ each with "energy" or quality w_β. It will be useful to divide the input and output energy fluxes that are consumed/secreted in metabolite β by $J^{\rm in}_\beta$ and $J^{\rm out}_\beta$ respectively. We define the fraction $f^{\rm out}_\beta$ of the *output energy* secreted as resource β by

$$J^{\rm out}_\beta \equiv f^{\rm out}_\beta J^{\rm out}. \qquad [2]$$

We can define corresponding mass fluxes by

$$\nu^{\rm out}_\beta \equiv J^{\rm out}_\beta / w_\beta \qquad [3]$$

and

$$\nu^{\rm in}_\beta \equiv J^{\rm in}_\beta / w_\beta \qquad [4]$$

In general, all these fluxes depend on the species under consideration and will carry an extra roman index i indicating the species.

We assume that a fixed quantity m_i of power per cell is required for maintenance of species i, and that the per-capita growth rate is proportional to the remaining energy flux $(J^{\rm growth} - m_i)$, with proportionality constant g_i. Under these assumptions, the time-evolution of the population size N_i of species i can be modeled using the equation

$$\frac{dN_i}{dt} = g_i N_i (J^{\rm growth}_i - m_i). \qquad [5]$$

We can model the resource dynamics by functions of the form

$$\frac{dR_\alpha}{dt} = h_\alpha(R_\alpha) - \sum_j N_j \nu^{\rm in}_{j\alpha} + \sum_j N_j \nu^{\rm out}_{j\alpha}, \qquad [6]$$

where the function h_α describes the resource dynamics in the absence of consumers. We can consider two kinds of dynamics: externally supplied and self-renewing. For externally supplied resources, we take a linearized form of the dynamics:

$$h^{\rm external}_\alpha(R_\alpha) = \kappa_\alpha - \tau_\alpha^{-1} R_\alpha \qquad [7]$$

while for self-renewing we take a logistic form for the dynamics

$$h^{\rm self-renewing}_\alpha(R_\alpha) = r_\alpha R_\alpha (K_\alpha - R_\alpha). \qquad [8]$$

In the present study, we only consider externally supplied resources.

These equations specify the general dynamics of all the models we consider. Metabolism is encoded in the relationship between input, output, and growth fluxes.

B. Input fluxes and output partitioning. We will now specify the form of the input fluxes $\nu^{\rm in}_\beta$ and the output partitioning $f^{\rm out}_\beta$. This involves specifying how an input resource is turned into an metabolic byproducts. To try to capture metabolic structure, we will divide the M resources into T classes (e.g. sugars, amino acids, etc.), each with M_A resources where $A = 1, \dots T$ and $\sum_A M_A = M$. We will be interested in capturing coarse metabolic structure (i.e. metabolizing sugars outputs carboxylic acids, etc). We will limit ourselves to considering strictly substitutable resources.

In all consumer resource models, we assume that

$$\nu^{\rm in}_{i\beta} = \sigma(c_{i\beta} R_\beta) \qquad [9]$$

where σ is a single-valued function encoding the relationship between resource supply levels and uptake rates. In the microbial context the consumer preferences $c_{i\alpha}$ can be interpreted as expression levels of transporters for each of the resources. We consider three kinds of response functions: Type-I, linear response functions where

$$\sigma_I(x) = x, \qquad [10]$$

a Type-II saturating Monod function,

$$\sigma_{II}(x) = \frac{x}{1+\frac{x}{K}} \qquad [11]$$

and a Type-III Hill or sigmoid-like function

$$\sigma_{III}(x) = \frac{x^n}{1+\left(\frac{x}{K}\right)^n}, \qquad [12]$$

where $n > 1$.

In all the simulations of this paper, we assume that resources independently contribute to the growth rate. We define a leakage fraction $0 \le l_\alpha \le 1$ for resource α such that

$$J_\alpha^{\text{out}} = l_\alpha J_\alpha^{\text{in}}. \qquad [13]$$

A direct consequence of energy conservation (Equation (1)) is that

$$J_i^{\text{growth}} = \sum_\alpha (1-l_\alpha) J_{i\alpha}^{\text{in}} = \sum_\alpha (1-l_\alpha) w_\alpha \sigma(c_{i\alpha} R_\alpha) \qquad [14]$$

Finally, we denote by $D_{\beta\alpha}$ the fraction of the output energy that is contained in metabolite β when a cell consumes α. Note that by definition $\sum_\beta D_{\beta\alpha} = 1$. These $D_{\beta\alpha}$ and l_α uniquely specify the metabolic model for independent resources and we can write all fluxes in terms of these quantities.

The total energy output in metabolite β is thus

$$J_{i\beta}^{\text{out}} = \sum_\alpha D_{\beta\alpha} l_\alpha J_{i\alpha}^{\text{in}} = \sum_\alpha D_{\beta\alpha} l_\alpha w_\alpha \sigma(c_{i\alpha} R_\alpha). \qquad [15]$$

This also yields

$$\nu_{i\beta}^{\text{out}} = \sum_\alpha D_{\beta\alpha} l_\alpha \frac{w_\alpha}{w_\beta} \sigma(c_{i\alpha} R_\alpha) \qquad [16]$$

We are now in position to write down the full dynamics in terms of these quantities:

$$\begin{aligned} \frac{dN_i}{dt} &= g_i N_i \left[\sum_\alpha (1-l_\alpha) w_\alpha \sigma(c_{i\alpha} R_\alpha) - m_i \right] \\ \frac{dR_\alpha}{dt} &= h_\alpha(R_\alpha) - \sum_j N_j \sigma(c_{j\alpha} R_\alpha) \\ &\quad + \sum_{j\beta} N_j \sigma(c_{j\beta} R_\beta) \left[D_{\alpha\beta} \frac{w_\beta}{w_\alpha} l_\beta \right] \end{aligned} \qquad [17]$$

Notice that when σ is Type-I (linear) and $l_\alpha = 0$ for all α (no leakage or byproducts), this reduces to MacArthur's original model (1).

C. Choosing consumer preferences. We will now choose consumer preferences $c_{i\alpha}$ as follows. We assume that each specialist family has a preference for one resource class A (where $A = 1 \dots F$) with $0 \le F \le T$, and we denote the consumer coefficients for this family by $c_{i\alpha}^A$. We will also consider generalists that have no preferences, with consumer coefficients $c_{i\alpha}^{\text{gen}}$. We will consider three kinds of models: one where the coefficients are drawn from Gaussian distributions, another where they are drawn from Gamma distributions (which ensure positivity of the coefficients), and finally a discrete, binary preference model.

C.1. Gaussian consumer preferences. The Gaussian model allows a continuous gradation of transporter expression levels. We assume that the variance is fixed to so that for all coefficients for all families

$$\langle (\delta c_{i\alpha}^A)^2 \rangle = \langle (\delta c_{i\alpha}^{\text{gen}})^2 \rangle = \frac{\sigma_c^2}{M}. \qquad [18]$$

In the generalist family, the mean is also the same for all resources, and is given by

$$\langle c_{i\alpha}^{\text{gen}} \rangle = \frac{\mu_c}{M}. \qquad [19]$$

The specialist families sample from a distribution with a larger mean for resources in their preferred class:

$$\langle c_{i\alpha}^A \rangle = \begin{cases} \frac{\mu_c}{M}\left[1 + \frac{M-M_A}{M_A} q_A\right], & \text{if } \alpha \in A \\ \frac{\mu_c}{M}(1-q_A), & \text{otherwise,} \end{cases} \qquad [20]$$

where M_A is the number of resources in class A, and q_A controls how much more species from family A prefer resources from class A.

We have put a factor of M in the denominators of the expressions for mean and variance, because the sums over $c_{i\alpha}$ in the dynamical equations (17) always give rise to terms with means $M\langle c_{i\alpha}\rangle$ or $S\langle c_{i\alpha}\rangle$ and variances $M\langle(\delta c_{i\alpha})^2\rangle$ or $S\langle(\delta c_{i\alpha})^2\rangle$. The factor of M allows us to keep σ_c, μ_c fixed when exploring the $M, S \to \infty$ limit in Section 5 below.

C.2. Gamma consumer preferences. We will also consider the case where consumer preferences are drawn from Gamma distributions, which guarantee that all coefficients will be positive. Since the Gamma distribution only has two parameters, it is fully determined once the mean and variance are specified. We parameterize the mean and variance for this model in the same way as for the Gaussian model.

C.3. Binary consumer preferences. In the binary model, there are only two possible expression levels for each transporter: a low level $\frac{c_0}{M}$ and a high level $\frac{c_0}{M} + c_1$. The elements of $c^A_{i\alpha}$ are given by

$$c^A_{i\alpha} = \frac{c_0}{M} + c_1 X_{i\alpha}, \quad [21]$$

where $X_{i\alpha}$ is a binary random variable that equals 1 with probability

$$p^A_{i\alpha} = \begin{cases} \frac{\mu_c}{Mc_1}\left[1 + \frac{M-M_A}{M_A} q_A\right], & \text{if } \alpha \in A \\ \frac{\mu_c}{Mc_1}(1-q_A), & \text{otherwise} \end{cases} \quad [22]$$

for the specialist families, and

$$p^{\text{gen}}_{i\alpha} = \frac{\mu_c}{Mc_1} \quad [23]$$

for the generalists.

Note that the mean of the distribution is $\langle c_{i\alpha}\rangle = p_{i\alpha}c_1 + \frac{c_0}{M}$, and the variance is $\langle(\delta c_{i\alpha})^2\rangle = p_{i\alpha}(1-p_{i\alpha})$. Both of these scale as $1/M$ when $M \to \infty$, just like the Gaussian and Gamma versions, as long as c_1, c_0 and μ_c are held fixed.

D. Constructing the metabolic matrix. We choose the metabolic matrix $D_{\alpha\beta}$ according to a three-tiered secretion model. The first tier contains a preferred class of byproducts, such as carboyxlic acids for fermentative and respiro-fermentative bacteria, which includes M_c members. The second contains byproducts of the same class as the input resource (when the input resource is not in the preferred byproduct class). For example, this could be attributed to the partial oxidation of sugars into sugar alcohols, or the antiporter behavior of various amino acid transporters. The third tier includes everything else. We encode this structure in $D_{\alpha\beta}$ by sampling each column of the matrix from a Dirichlet distribution with concentration parameters $d_{\alpha\beta}$ that depend on the byproduct tier, so that on average a fraction f_c of the secreted flux goes to the first tier, while a fraction f_s goes to the second tier, and the rest goes to the third:

$$d_{\alpha\beta} = \begin{cases} d_0 \frac{f_c+f_s}{M_c}, & \text{if } \alpha = c \\ d_0 \frac{1-f_c-f_s}{M-M_c}, & \text{if } \alpha \neq c \text{ and } \beta = c \\ d_0 \frac{f_s}{M_{A(\beta)}}, & \text{if } \alpha,\beta \neq c \text{ and } A(\alpha) = A(\beta) \\ d_0 \frac{1-f_s-f_c}{M-M_{A(\beta)-M_c}}, & \text{if } \alpha,\beta \neq c \text{ and } A(\alpha) \neq A(\beta). \end{cases} \quad [24]$$

The parameter d_0 controls the randomness of the partitioning, ranging from deterministic partitioning when $d_0 \to 0$, to maximally stochastic partitioning (with each input resource having just one randomly chosen output resource) as $d_0 \to 1$.

The mean of the Dirichlet distribution is always equal to $1/M$, and the variance under this parameterization also scales as $1/M$ when the f's and d_0 are held fixed. The sampling of $D_{\alpha\beta}$ thus following the same scaling behavior as our scheme for the consumer matrices in the $M, S \to \infty$ limit of Section 5.

2. Simulation and data analysis

A. The Community Simulator. We implemented the above modeling framework in a Python package called "Community Simulator," which can be downloaded and installed from https://github.com/Emergent-Behaviors-in-Biology/community-simulator. Once this package is installed, the data can be downloaded from https://github.com/Emergent-Behaviors-in-Biology/crossfeeding-transition, and the accompanying Jupyter notebook can be used to regenerate all the figures. The one exception is the energy flux network figure, which was generated in MATLAB using a file exported from the notebook. The repository also contains a sample MATLAB script for loading and visualizing the network file.

Community Simulator is designed to run dynamics on multiple communities in parallel, inspired by the parallel experiments commonly performed with 96-well plates. The central object of the package is a `Community` class, whose instances are initialized by specifying the initial population sizes and resource concentrations for each parallel "well," along with the functions and parameters that define the population dynamics. This class contains two core methods. `Propagate(T)` sends each community to a separate CPU (for however many CPU's are available), runs the given population dynamics for a time T using the SciPy function `odeint`, and updates the population sizes and resource concentrations in each well to the time-evolved values. `Passage(f)` initializes a fresh set of wells by adding a fraction $f_{\mu\nu}$ of the contents of each old well ν to each new well μ. (Fresh media can also be added at this point, but this feature was not relevant for the current work). The resulting values of N_i are converted from arbitrary concentration units to actual population sizes using a specified scale factor, and then integer population sizes are obtained by multinomial sampling based on these values.

The Community Simulator package also contains a set of scripts for generating models and randomly sampling parameters. `MakeConsumerDynamics` and `MakeResourceDynamics` from the `usertools` module take a dictionary of assumptions concerning the response type, metabolic regulation and resource replenishment, and generate the corresponding functions for dN_i/dt and dR_α/dt. The function `MakeMatrices`, from the same module, randomly samples the consumer matrix $c_{i\alpha}$ and the metabolic matrix $D_{\alpha\beta}$.

B. Simulation Details. For this paper, we generated a binary consumer matrix with $c_0 = 0.01$, $c_1 = 1$ and $\mu_c = 10$, and a metabolic matrix with $d_0 = 0.2$. This matrix defined a regional pool of $S = 200$ species, consuming $M = 100$ possible resource types. We used only one family and one resource class in constructing the $c_{i\alpha}$ and $D_{\alpha\beta}$ matrices (but arbitrarily assigned each resource and each species to one of four categories, as a null model for comparison with future structured simulations). We set $w_\alpha = g_i = 1$ for all i and α, and set the l_α for all resources equal to each other. For the multinomial sampling described above, we chose the scale factor so that $N_i = 1$ corresponds to a population of 10^6 cells. We generated dynamics with Type-I response and no regulation. Resource type 0 was externally supplied with flux κ_0, and all the other κ_α's were set to zero.

To simulate stochastic colonization, we initialized each of 10 wells with 100 randomly chosen species from the regional pool, with a population size of 10^6 cells per species per well. We propagated each well under Equations (17) for a time $\Delta t = 11,500$, which is much longer than the maximum time required to relax to the steady state for any of the parameter regimes sampled. We used the `Passage` method with $f_{\mu\nu} = \delta_{\mu\nu}$ to periodically eliminate species whose populations became too small. For the large steady-state population sizes we consider here ($\sim 10^4 - 10^9$, see S4 Fig.), the multinomial sampling eliminates species whose populations are heading for extinction while minimally perturbing the dynamics of the survivors. We passaged after every 5 time units of propagation from the beginning of the simulation up to time $t = 500$, then every 100 time units until time $t = 1,500$, and finally every 1,000 time units up to the final time $t = 11,500$.

The timeseries shown in Figure 1E was generated under these assumptions, with $w_0\kappa_0 = 500$.

We propagated these 10 initial states using this procedure for 100 different combinations of externally supplied energy flux $w_0\kappa_0$ and leakage fraction l, with 10 $w_0\kappa_0$ values evenly spaced on a logarithmic scale from 10 to 100, and 10 l values evenly spaced from 0 to 0.9. Figure 2 of the main text shows the mean richness over the 10 parallel wells for each combination of $w_0\kappa_0$ and l. The richness is defined as the number of species with non-zero abundance at the end of the simulation.

We focused on three representative examples for further analysis:

1. **Syntrophy-Limited**: $w_0\kappa_0 = 1000$, $\langle l_\alpha \rangle = 0.1$

2. **Energy-Limited**: $w_0\kappa_0 = 28$, $\langle l_\alpha \rangle = 0.6$

3. **Similarity-Limited**: $w_0\kappa_0 = 1000$, $\langle l_\alpha \rangle = 0.9$.

The rank-abundance plots in Figure 2 of the main text show the population sizes in all 10 wells from each of these examples, after normalizing them by the total biomass $\sum_i N_i$. The plots were truncated at a relative abundance of 0.5% for clarity. Rank-abundance plots for these same three examples in absolute units with no truncation can be found in S4 Fig..

C. Susceptibilities. One important property of an ecosystem is its sensitivity to changes in environmental conditions. Figure 3 of the main text quantifies this sensitivity in terms of a set of susceptibilities, defined by

$$\chi_{\alpha\beta} \equiv \frac{\partial \bar{R}_\alpha}{\partial \kappa_\beta} \qquad [25]$$

$$\eta_{i\beta} \equiv \frac{\partial \bar{N}_i}{\partial \kappa_\beta} \qquad [26]$$

where $\bar{N}_i$, $\bar{R}_\alpha$ are the steady-state consumer populations and resource concentrations, respectively.

For the case of externally supplied resources and Type-I growth, setting Equations (17) equal to zero and differentiating with respect to κ_β yields:

$$0 = \sum_\alpha (1 - l_\alpha) w_\alpha c_{i\alpha} \chi_{\alpha\beta} \qquad [27]$$

$$-\tau_\alpha^{-1}\delta_{\alpha\beta} = \sum_\gamma \left(\sum_j c_{j\gamma} N_j \left[D_{\alpha\gamma} \frac{w_\gamma}{w_\alpha} l_\gamma - \delta_{\gamma\alpha} \right] - \tau_\alpha^{-1}\delta_{\gamma\alpha} \right) \times \chi_{\gamma\beta} + \sum_{j\gamma} c_{j\gamma} \left[D_{\alpha\gamma} \frac{w_\gamma}{w_\alpha} l_\gamma - \delta_{\gamma\alpha} \right] R_\gamma \eta_{j\beta} \qquad [28]$$

The last equation can be reorganized as

$$
\begin{aligned}
-\tau_\alpha^{-1}\delta_{\alpha\beta} = \sum_\gamma \Bigg(\sum_j c_{j\gamma} N_j \left[D_{\alpha\gamma}\frac{w_\gamma}{w_\alpha} l_\gamma - \delta_{\gamma\alpha}\right] \\
- \tau_\alpha^{-1}\delta_{\gamma\alpha}\Bigg)\chi_{\gamma\beta} \\
+ \sum_{j\gamma} c_{j\gamma}\left[D_{\alpha\gamma}\frac{w_\gamma}{w_\alpha} l_\gamma - \delta_{\gamma\alpha}\right] R_\gamma \eta_{j\beta}
\end{aligned}
\qquad [29]
$$

For each value of β, this system of linear equations can be solved for $\chi_{\gamma\beta}$ and $\eta_{j\beta}$ by simply inverting a matrix (once the terms corresponding to extinct species have been removed).

The histograms of Figure 3D in the main text contain the diagonal elements $\chi_{\alpha\alpha}$ for all resources except for the one supplied externally ($\alpha = 0$), which might be expected to behave somewhat differently. The $\chi_{\alpha\alpha}$ values from all 10 parallel communities are included in the histogram. We generated one histogram for the similarity-limited regime, and one for the energy-limited regime, using the examples defined in Section B above.

D. Niche Overlap. To find out what controls the diversity of the diverse regime, we varied the niche overlap, which quantifies the similarity among consumer preferences within the regional species pool. We did this by holding μ_c fixed, and varying c_1 from its original value of 1 down to a minimum value of 0.12. For each value of c_1, we generated 10 $c_{i\alpha}$ matrices, which each defined a regional pool of 200 species. We then repeated the procedure of Section B above for each of these regional pools: initializing 10 wells with 100 species and running them to the steady state with the same sequence of propagation and passage steps. The final richness of each community is plotted in Figure 4 of the main text as a translucent point, such that more common richness values are darker. We included all three examples defined at the end of Section B in the plot, and colored both examples from the resource-limited regime blue, while the diverse regime was colored red.

E. Beta Diversity. To examine the beta diversity patterns in each regime, we initialized 200 wells with 100 randomly chosen species from the regional pool of 200 species, and propagated them to steady state following Section B under the three different choices of $w_0\kappa_0$ and l listed at the end of that section. To visualize the variation among these communities, we used the Python package scikit-learn (2) to compute the first two principle components of the set of composition vectors in each regime. We then projected the compositions onto the plane spanned by these vectors, and generated a scatter plot of the results. We also computed the percentage of the total variance accounted for by each of these two principal components, and indicated the value in parentheses on each axis.

F. Data Format. The output of all the simulations was saved to a set of Microsoft Excel spreadsheets, which can be easily imported into Python for analysis using the Pandas package. Each simulation generated four files: final consumer populations (‘`Consumers`’), final resource concentrations (‘`Resources`’), a metadata summary (‘`Parameters`’), and initial conditions (‘`Initial_State`’). The $c_{i\alpha}$ and $D_{\alpha\beta}$ matrices as well as the m_i and w_α were pickled into a binary file (‘`Realization`’). The file names also include the date on which the data was generated, and a task ID when multiple files were generated on the same day.

The first column in the consumer and resource tables is the index of the simulation run. The second and third columns of the consumer file are the family ID and species ID, respectively. In the resource file, these columns contain the class ID and resource ID. The remaining columns contain the populations/concentrations for each well. The consumer populations are in units of 10^6 cells.

All the parameters that change between runs are included in the metadata file (‘`Parameters`’). The first column of this file is the simulation run index, corresponding to the index in the consumer and resource files.

The initial conditions file contains the initial population sizes for each of the wells.

3. Robustness of qualitative results

We tested the robustness of our qualitative results by modifying the modeling assumptions in five ways. We have given each way a descriptive name, which can be used to look up the raw data files from the supplemental data folder using the `file_list.csv` table:

- `main_dataset` is the data from the main text
- `type_II` uses a Type II functional response, with $K = 20$.
- `dense_metabolism` has a dense metabolic matrix with $d_0 = 0.001$.
- `randomness` adds (quenched) random variation to w_α and l_α, with standard deviations 0.1 and 0.03, respectively.
- `Gaussian_sampling` samples the $c_{i\alpha}$’s from Gaussian distributions, with the same mean 0.11 and standard deviation 0.3 as the binary matrix used in the main text.

- `Gamma_sampling` samples the $c_{i\alpha}$'s from Gamma distributions, with the same mean and variance.

The following sections describe each of these choices in more detail. S1 Fig., S4 Fig., S5 Fig. and S6 Fig. show the key plots from the main text along with the new versions generated under all these modified assumptions. S2 Fig. and S3 Fig. display another diversity measure not discussed in the main text: the Simpson Diversity (S. D.). This is defined analogously to the "effective number of resources consumed" presented in Equation (6) of the main text:

$$\text{S.D.} = \left[\sum_i \left(\frac{N_i}{N}\right)^2\right]^{-1} \qquad [30]$$

where $N \equiv \sum_i N_i$. As discussed in the main text in connection with resource fluxes, this quantity approaches 1 when there is one large $N_i \approx N$ and all the other populations are very small. It approaches the number of species (i.e., the richness) as the biomass distribution becomes more uniform.

A. Type-II Growth. We chose the Monod parameter $K = 20$ in the Type-II growth simulations in order to ensure that at least one species would survive in the steady state in all simulations. The maximum possible incoming energy flux in the Type-II model is equal to $0.1K$ when $w_\alpha = 1$ and $l = 0.9$, and this must exceed $m_i \approx 1$ for a species to survive. $K = 20$ provides a maximum flux of 2 in this case.

B. Metabolic Matrices. The metabolic matrices $D_{\beta\alpha}$ are plotted in Figure 3 of the main text for `main_dataset` and `dense_metabolism` (all other simulations use the same metabolic parameters as `main_dataset`). We see that $d_0 = 0.2$ leads to a very sparse matrix, with only a few secreted byproducts per input resource, while the secretion fractions for $d_0 = 0.001$ are much more uniform.

C. Randomness in w_α and l_α. To relax the assumption of all the w_α's and l_α's being equal, we sampled these two vectors from Gaussian distributions. We chose the standard deviations of the distributions to be small enough that both quantities would almost always be positive, and l_α would remain less than 1.

D. Gaussian and Gamma Sampling. Sampling consumer preferences from the continuous Gaussian and Gamma distributions makes the differential equations much more stiff than in the binary case. To ensure stable operation of the integrator, we "passaged" the cells every 0.1 time units. Each call of the "passage" method zeros out small negative values of resource concentration or consumer population that arise because of numerical error, in addition to setting small consumer populations to zero. This high frequency of passaging made the simulation more computationally intensive, so we only propagated these simulations for 200 time units. We computed the root-mean-square difference between the per-capita growth rates $(1/N_i)(dN_i/dt)$ and zero to check whether the simulations had converged. We found that all of them had acceptably converged, except for some of the runs at $w_0\kappa_0 < 100$ in the Gaussian case. The Gaussian model is unphysical, because almost half of the consumer preferences are less than 0 for these simulations, and so we decided not to spend more computation time in pursuit of convergence.

4. Quantification of Nestedness

Almeida-Neto *et al.* have introduced a quantitative measure of nestedness, called the "Nestedness metric based on Overlap and Decreasing Fill," or NODF (3). S7 Fig. shows how the NODF depends on the relative abundance threshold for the Tara Oceans data, as compared with two null models taken from the Earth Microbiome Project analysis (4). Null Model 1 keeps the richness of each sample the same while randomly altering the identities of the surviving species. It tells us how much nestedness we should expect by chance from a set of samples with the given levels of diversity, in the absence of any ecological mechanisms. Null Model 2 keeps the prevalence of each species the same while randomly assigning it to different samples. This procedure generates another well-defined family of random matrices with similar bulk statistics to the original data, but lacks the operational interpretation of Null Model 1. For this reason, the comparison with Null Model 1 is more meaningful, but we include Null Model 2 for completeness.

For each of the null models and each value of the relative abundance threshold, we generated 100 random permutations of the data matrix and computed the mean and standard deviation of their NODF scores. We found that the actual nestedness exceeds that of Null Model 1 by at least 20 standard deviations for all values of the threshold. For the relative abundance threshold of 0.5% employed in Figure 7 of the main text, the true NODF also exceeds Null Model 2 by 5.8 standard deviations.

The figure also shows histograms generated with the two null models from the simulation data of Figure 6A. The actual NODF (=0.46) is more than 100 standard deviations above the mean nestedness for both models.

To compute the NODF, we employed the following algorithm, which is implemented in the Community Simulator package (in the `analysis` module). Let n be the number of columns, and m the number of rows in a matrix A. Let D_c be an $n \times n$ matrix, such that $(D_c)_{ij} = 1$ if the sum of column i is greater than the sum of column j, and 0 otherwise. Similarly, let D_r be an $m \times m$ matrix such that $(D_r)_{ij} = 1$ if the sum of row j is greater than the sum of row i, and zero otherwise. Let B be the row-normalized matrix, where each row of A has been divided by the sum over the row. And let C be the column-normalized matrix, where each column has been normalized by the sum over the column. Then the NODF of the matrix A is

$$\mathrm{NODF} = 2\frac{\mathrm{Tr}(A^T D_r B) + \mathrm{Tr}(A D_c C^T)}{n(n-1) + m(m-1)} \quad [31]$$

where Tr represents the trace operation.

5. Numerical Evidence of a Phase Transition

A phase transition in physics is characterized by a discontinuous change in the value of an observable or its derivative as an intensive parameter is varied, in the "thermodynamic limit" of infinite system size. In an ecological context, the analog to system size is the number of possible resource types M, or the initial number of species S. Several recent works have explored the analytic computations that become tractable in the $M, S \to \infty$ limit of various models, while $\gamma \equiv M/S$ remains constant (when the model is resource-explicit) (5–8). Taking this limit requires several decisions about how to scale the rest of the parameters. Our sampling scheme for the $c_{i\alpha}$ and $D_{\alpha\beta}$ matrices, described in Section 2, follows the canonical strategy for studying spin glasses, where the random coupling parameters J_{ij} are chosen such that the mean and variance are both proportional to $1/M$ (9). The maintenance costs m_i, on the other hand, are sampled from the same distribution regardless of the value of M. Finally, we note that the total amount of energy $w_0\kappa_0$ supplied to the system is an "extensive" parameter, and that the scaling analysis should be performed with the corresponding "intensive" parameter $w_0\kappa_0/M$ held fixed.

S8 Fig. shows how the consumer richness scales with M for each of the three examples discussed in the main text. The first two examples come from the resource-limited regime. The "syntrophy-limited" example has $w_0\kappa_0/M = 10, l = 0.1$, while the "energy-limited" example has $w_0\kappa_0/M = 0.28, l = 0.6$. The third, "similarity-limited" example comes from the diverse regime, with $w_0\kappa_0/M = 10, l = 0.9$. The richness appears to scale like M^α with exponent $\alpha < 1$ for the resource-limited examples, and $\alpha = 1$ for the diverse example. If this scaling holds asymptotically as $M \to \infty$, then the system exhibits a true phase transition, with the normalized richness (richness/M) vanishing in the resource-limited regime, while remaining finite in the diverse regime. The gray line in the right-hand panel illustrates what this would look like, with a discontinuity in the derivative of the normalized richness as a function of l or $w_0\kappa_0/M$. This evidence is by no means conclusive, since we only have access to a single decade of M values. To reach three decades of M values would require solving 40,000 coupled ODE's involving matrices $c_{i\alpha}$ and $D_{\alpha\beta}$ with size $20,000 \times 20,000$. Each matrix would thus have 4×10^8 entries, corresponding to 50 MB of memory for binary entries or 800 MB of memory for floating-point entries. This computation is feasible but non-trivial, demanding significantly more attention to how the matrix multiplications are implemented and how the matrices are passed around in memory. We are currently working on an update to the Community Simulator package that implements the core computations in PyTorch, which enables GPU acceleration of matrix multiplication and may allow for calculations on this scale.

For completeness, S9 Fig. shows how four other natural observables scale with system size. These are the Simpson and Shannon diversity of the steady-state consumer and resource abundances (10). To compute these quantities, one first obtains relative abundances $f_i = N_i/\sum_i N_i$ and $f_\alpha = R_\alpha/\sum_\alpha R_\alpha$. In terms of these fractions, the Simpson diversity is

$$D_{\mathrm{Sim}} = \left(\sum_i f_i^2\right)^{-1} \quad [32]$$

and is related to the Inverse Participation Ratio commonly analyzed in spin glass problems, while the Shannon diversity is

$$D_{\mathrm{Sh}} = \exp\left(-\sum_i f_i \ln f_i\right), \quad [33]$$

and is simply the exponential of the Shannon entropy of the distribution (where the sum is taken only over the species with nonzero abundance). Both of these quantities are equal to 1 in the limit where a single type dominates the distribution, and equal the total number of surviving types when all the types have the same abundance. The Simpson and Shannon diversity of the consumers appear to saturate at a finite values in the large M limit of the resource-limited regime. When measured in these ways, the diversity of this regime thus appears to be insensitive to the size of the regional species pool and to the number of possible resource types, and is controlled by the energy supply, leakage fraction, and probably also the sparsity of the $D_{\alpha\beta}$ matrix.

References

1. MacArthur R (1970) Species Packing and Competitive Equilibrium for Many Species. *Theoretical Population Biology* 1:1.
2. Pedregosa F, et al. (2011) Scikit-learn: Machine learning in Python. *Journal of Machine Learning Research* 12:2825–2830.
3. Almeida-Neto M, Guimaraes P, Guimaraes Jr PR, Loyola RD, Ulrich W (2008) A consistent metric for nestedness analysis in ecological systems: reconciling concept and measurement. *Oikos* 117(8):1227.
4. Thompson LR, et al. (2017) A communal catalogue reveals Earth's multiscale microbial diversity. *Nature* 551:457.
5. Advani M, Bunin G, Mehta P (2018) Statistical physics of community ecology: a cavity solution to MacArthur's consumer resource model. *Journal of Statistical Mechanics* p. 033406.

6. Tikhonov M, Monasson R (2017) Collective phase in resource competition in a highly diverse ecosystem. *Physical Review Letters* 118:048103.
7. Bunin G (2017) Ecological communities with Lotka-Volterra dynamics. *Physical Review E* 95:042414.
8. Barbier M, Arnoldi JF, Bunin G, Loreau M (2018) Generic assembly patterns in complex ecological communities. *Proceedings of the National Academy of Sciences*.
9. Nishimori H (2001) *Statistical Physics of Spin Glasses and Information Processing*. (Oxford University Press, New York, NY).
10. Tuomisto H (2010) A consistent terminology for quantifying species diversity? Yes, it does exist. *Oecologia* 164:853.
