# Supplementary material for: Available energy fluxes drive a transition in the diversity, stability, and functional structure of microbial communities
Source: PLoS Comput Biol. 2019 Feb 5;15(2):e1006793. doi: 10.1371/journal.pcbi.1006793 (PMC6386421; doi:10.1371/journal.pcbi.1006793)
Supplement: S1 Fig — (PDF) [file pcbi.1006793.s002.pdf]

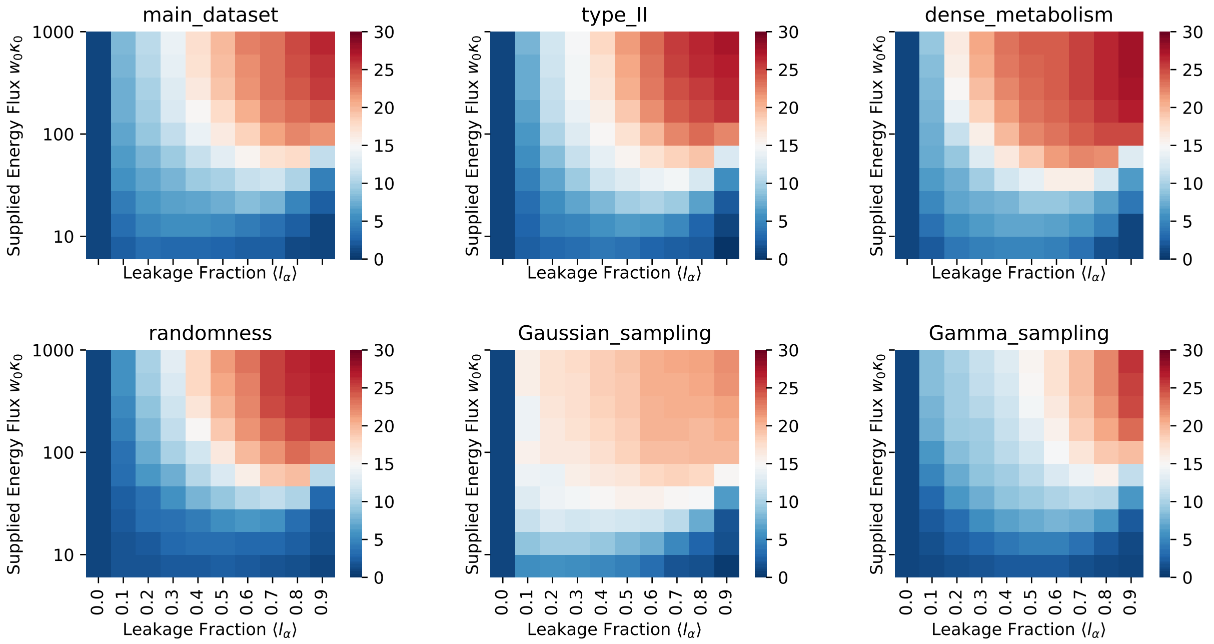

Figure S1: **Richness vs. $w_0\kappa_0$ and $\langle l_\alpha \rangle$ under different modeling choices.** We generated 200 species, initialized 10 communities of 100 species each from this pool, and ran the dynamics to steady state under different combinations of $w_0\kappa_0$ and $\langle l_\alpha \rangle$, for each of the six model choices listed at the beginning of Supporting Text Section 3. The color of each square indicates the mean number of non-extinct species at the end of the simulation, over all 10 communities at each combination of $w_0\kappa_0$ and $\langle l_\alpha \rangle$.
