# Supplementary material for: Available energy fluxes drive a transition in the diversity, stability, and functional structure of microbial communities
Source: PLoS Comput Biol. 2019 Feb 5;15(2):e1006793. doi: 10.1371/journal.pcbi.1006793 (PMC6386421; doi:10.1371/journal.pcbi.1006793)
Supplement: S2 Fig — (PDF) [file pcbi.1006793.s003.pdf]

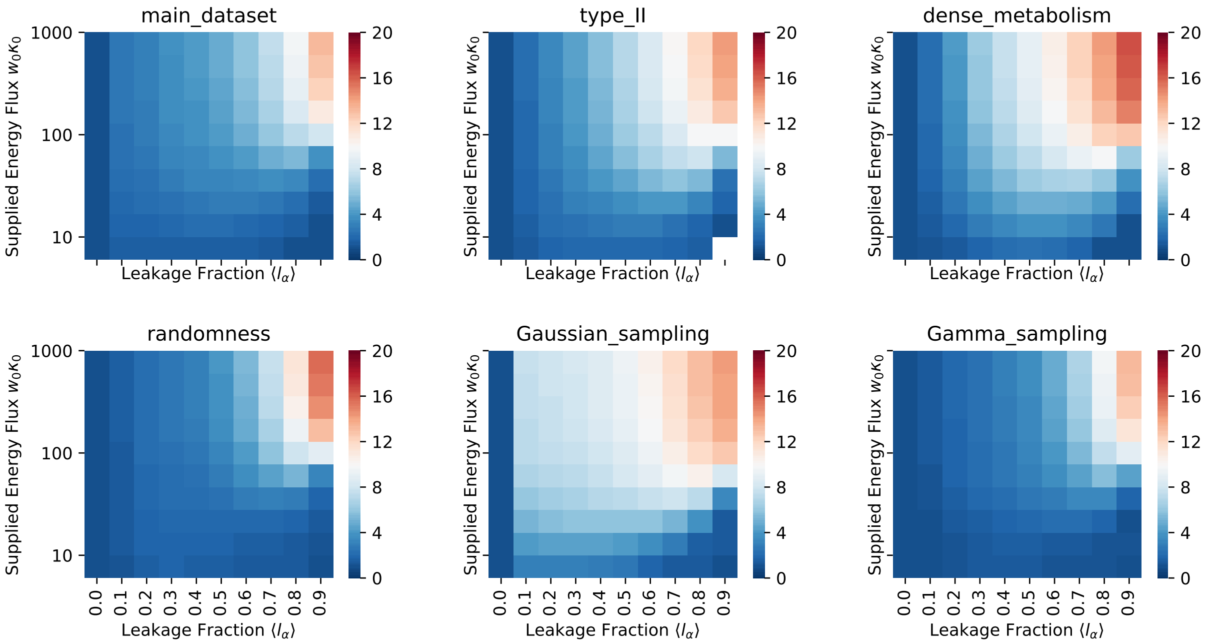

Figure S2: **Simpson Diversity vs. $w_0\kappa_0$ and $\langle l_\alpha \rangle$ under different modeling choices.** Simpson Diversity was computed according to Equation (30) of the Supporting Text, for each of the six model choices listed at the beginning of Supporting Text Section 3.
