# Supplementary material for: Available energy fluxes drive a transition in the diversity, stability, and functional structure of microbial communities
Source: PLoS Comput Biol. 2019 Feb 5;15(2):e1006793. doi: 10.1371/journal.pcbi.1006793 (PMC6386421; doi:10.1371/journal.pcbi.1006793)
Supplement: S3 Fig — (PDF) [file pcbi.1006793.s004.pdf]

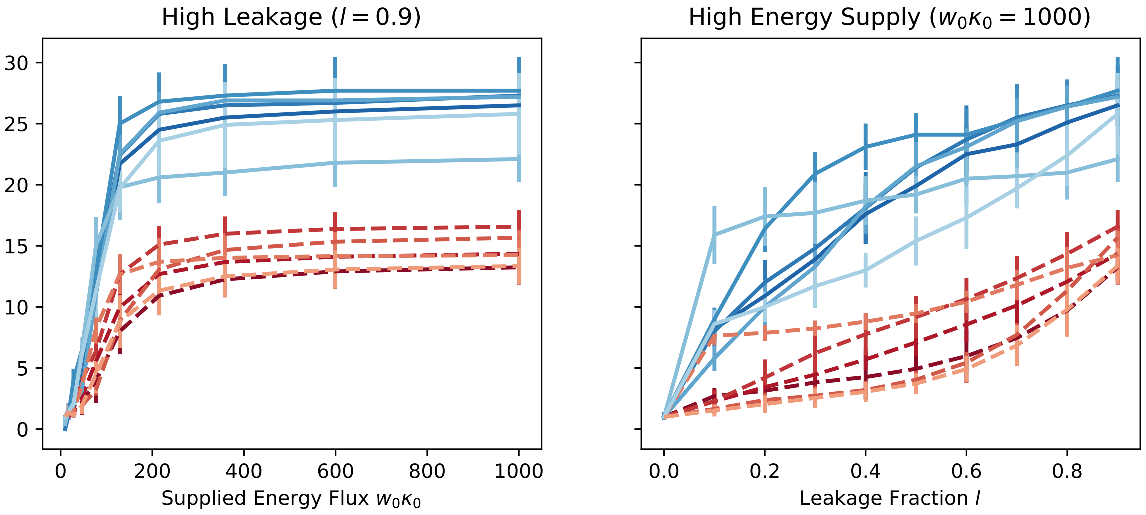

Figure S3: **Richness (blue solid) and Simpson Diversity (red dotted) for cuts through the heat map.** All six modeling choices described in Supporting Text Section 3 are plotted, going from dark to light in the order listed in the text. Error bars are plus and minus one standard deviation, where the standard deviation is computed over the 10 parallel communities at each set of parameter values.
