# Supplementary material for: Available energy fluxes drive a transition in the diversity, stability, and functional structure of microbial communities
Source: PLoS Comput Biol. 2019 Feb 5;15(2):e1006793. doi: 10.1371/journal.pcbi.1006793 (PMC6386421; doi:10.1371/journal.pcbi.1006793)
Supplement: S4 Fig — (PDF) [file pcbi.1006793.s005.pdf]

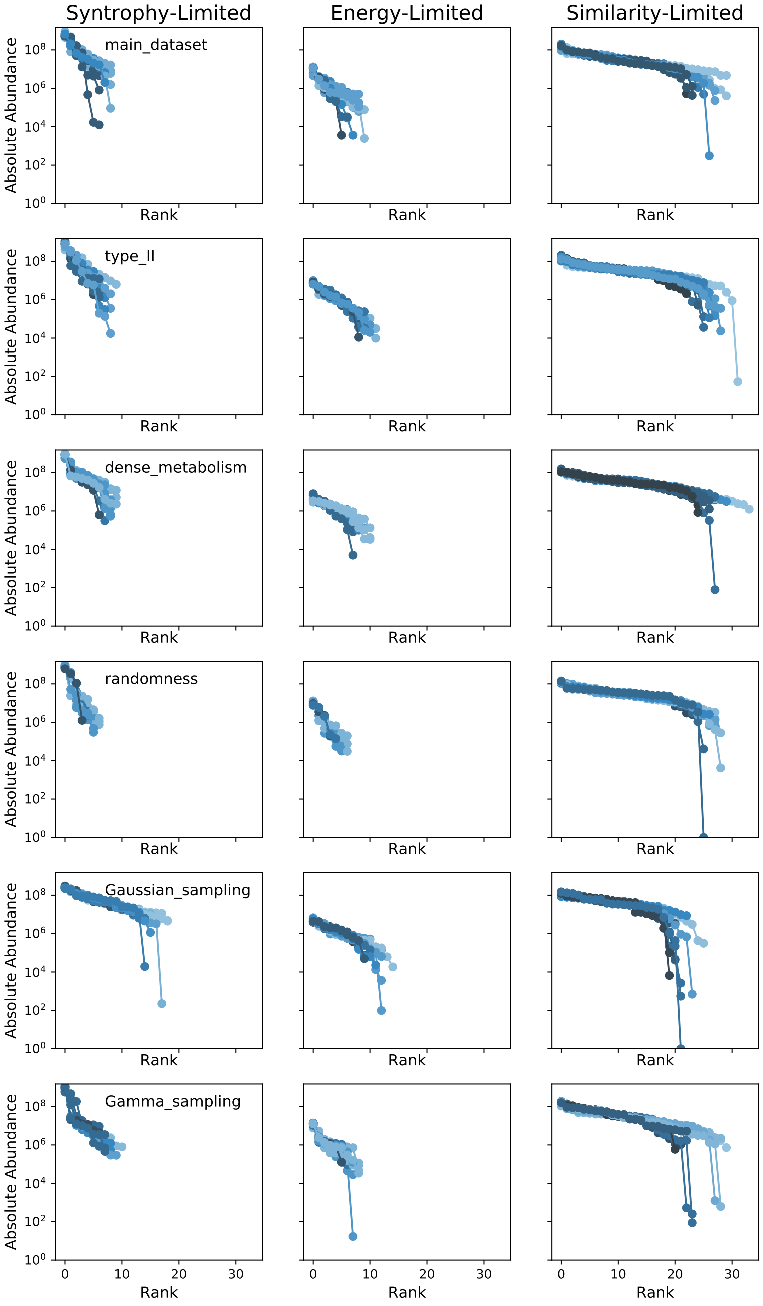

Figure S4: **Rank-abundance curves for three representative examples.** The abundance is plotted in absolute number of cells, as opposed to the relative abundance of the main text, and includes all species, with no truncation. The lower limit of the vertical axis is set to 1 cell, which is the smallest possible population size once the Passage method described in Supporting Text Section 2A has generated integer population values. The three examples are the same ones highlighted in Figure 2 of the main text, and listed in Supporting Text Section 2B.
