# Supplementary material for: Available energy fluxes drive a transition in the diversity, stability, and functional structure of microbial communities
Source: PLoS Comput Biol. 2019 Feb 5;15(2):e1006793. doi: 10.1371/journal.pcbi.1006793 (PMC6386421; doi:10.1371/journal.pcbi.1006793)
Supplement: S5 Fig — (PDF) [file pcbi.1006793.s006.pdf]

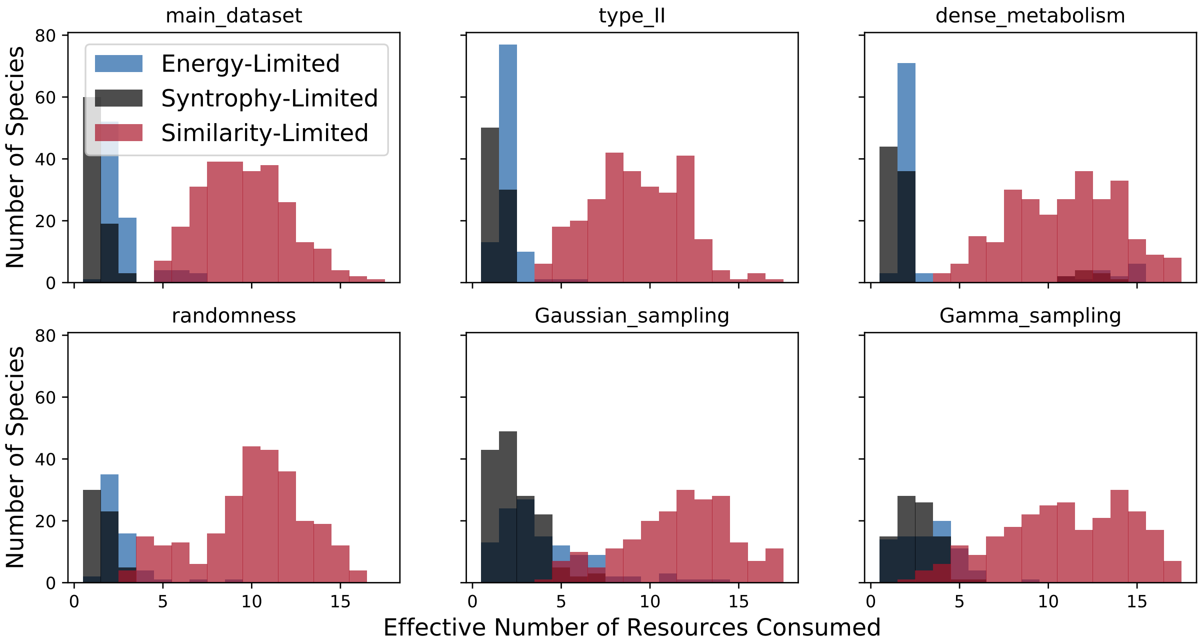

Figure S5: **Effective number of resources consumed under different modeling choices.** The effective number of resources consumed M_i^{eff} is computed as described in the main text for the three examples highlighted in Figure 2, for each of the six model choices listed at the beginning of Supporting Text Section 3. The y axis indicates the total number of species falling into each bin from the combination of all 10 parallel communities in each example.
