# Supplementary material for: Available energy fluxes drive a transition in the diversity, stability, and functional structure of microbial communities
Source: PLoS Comput Biol. 2019 Feb 5;15(2):e1006793. doi: 10.1371/journal.pcbi.1006793 (PMC6386421; doi:10.1371/journal.pcbi.1006793)
Supplement: S6 Fig — (PDF) [file pcbi.1006793.s007.pdf]

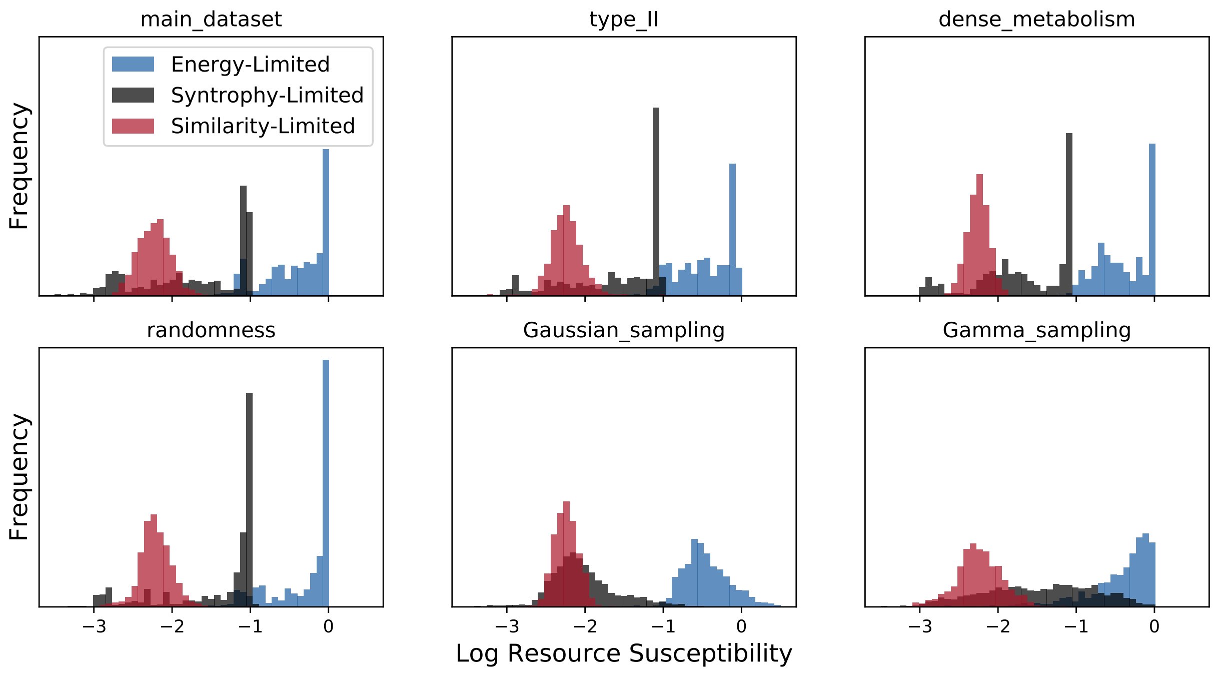

Figure S6: **Resource susceptibility.** Histograms of the logarithm $\log_{10} \partial \bar{R}_\alpha / \partial \kappa_\alpha$ are plotted for the three examples highlighted in Figure 2, for each of the six model choices listed at the beginning of Supporting Text Section 3. The y axis indicates the total number of species falling into each bin from the combination of all resources for all 10 parallel communities in each example, excepting the externally supplied resource $\alpha = 0$.
