# Supplementary material for: Available energy fluxes drive a transition in the diversity, stability, and functional structure of microbial communities
Source: PLoS Comput Biol. 2019 Feb 5;15(2):e1006793. doi: 10.1371/journal.pcbi.1006793 (PMC6386421; doi:10.1371/journal.pcbi.1006793)
Supplement: S7 Fig — (PDF) [file pcbi.1006793.s008.pdf]

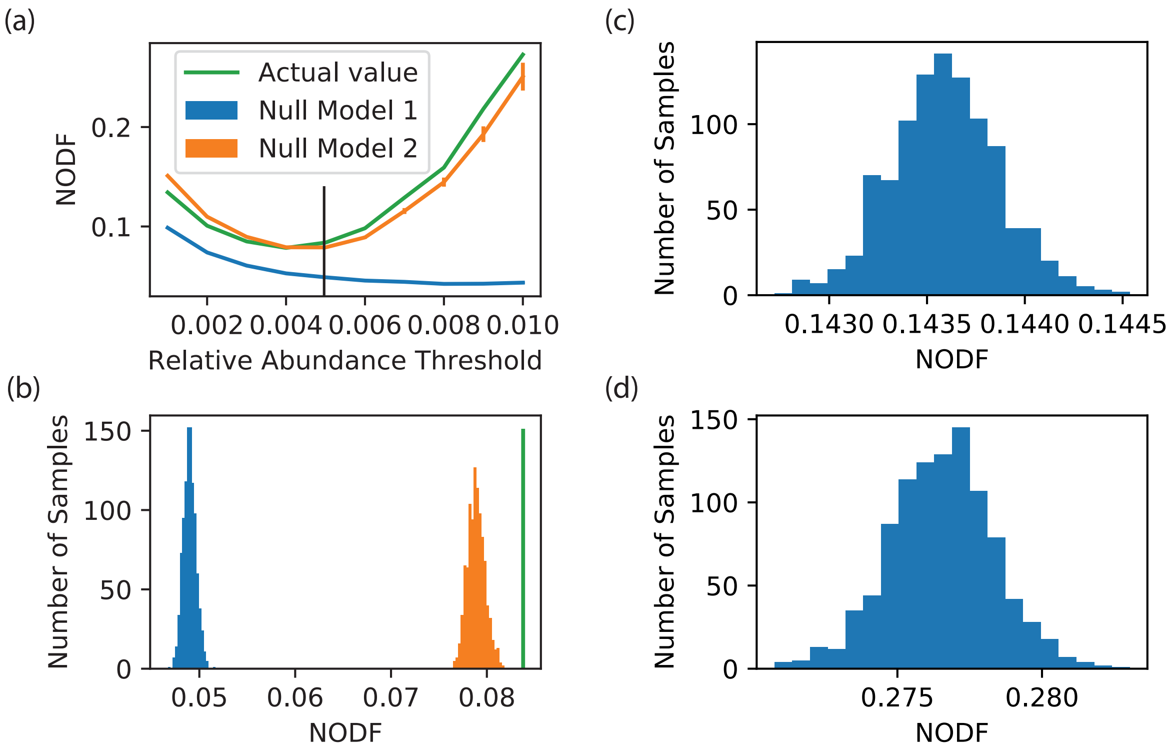

Figure S7: **Quantification of nestedness**. (a) Nestedness as quantified by NODF of the Tara Oceans data, for different values of the relative abundance threshold. See Supporting Text Section 3E for details on this metric. Also shown are the mean NODF over 100 samples from two null models, with error bars representing ± 1 standard deviation. Null Model 1 keeps the richness of each sample the same while randomly altering the identities of the surviving species. Null Model 2 keeps the prevalence of each species the same while randomly assigning it to different samples. (b) Histograms of 1000 samples from each null model at the relative abundance threshold of 0.005 used in the main text, and indicated with a black line in the first panel. (c), (d) Null models for comparison with the NODF value of 0.46 obtained for the simulation data. The two panels show histograms of 1,000 samples from null models 1 and 2, respectively.
