# Supplementary material for: Available energy fluxes drive a transition in the diversity, stability, and functional structure of microbial communities
Source: PLoS Comput Biol. 2019 Feb 5;15(2):e1006793. doi: 10.1371/journal.pcbi.1006793 (PMC6386421; doi:10.1371/journal.pcbi.1006793)
Supplement: S8 Fig — (PDF) [file pcbi.1006793.s009.pdf]

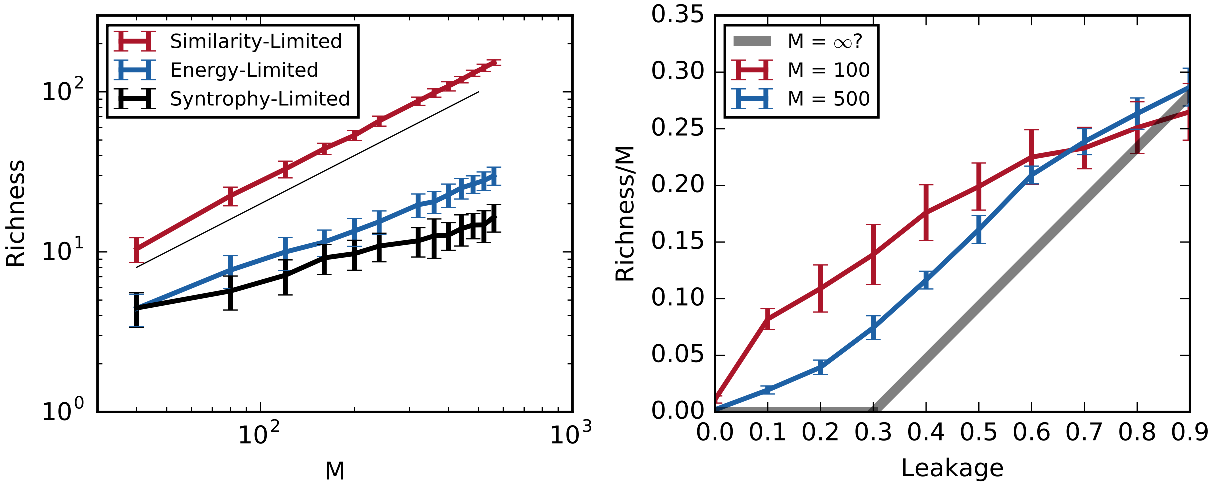

Figure S8: **Scaling of consumer richness with system size**. Left: Community richness in the three examples highlighted in Figure 2, as a function of the number of resource types M (with the rest of the parameters scaled as described in Supporting Text Section 1). The thin black line shows the slope corresponding to a linear scaling with M. Error bars are standard deviations over 100 samples per point. Right: Normalized richness as a function of leakage fraction at high energy supply $w_0\kappa_0/M = 10$. The top red line comes from the simulations of Figure 2 of the main text ($M = 100$), and the middle blue line comes from new simulations with $M = 500$. The bottom gray line illustrates a possible $M \to \infty$ limit, which would correspond to a continuous phase transition. Error bars are standard deviations over 10 samples per point.
