# Supplementary material for: Available energy fluxes drive a transition in the diversity, stability, and functional structure of microbial communities
Source: PLoS Comput Biol. 2019 Feb 5;15(2):e1006793. doi: 10.1371/journal.pcbi.1006793 (PMC6386421; doi:10.1371/journal.pcbi.1006793)
Supplement: S9 Fig — (PDF) [file pcbi.1006793.s010.pdf]

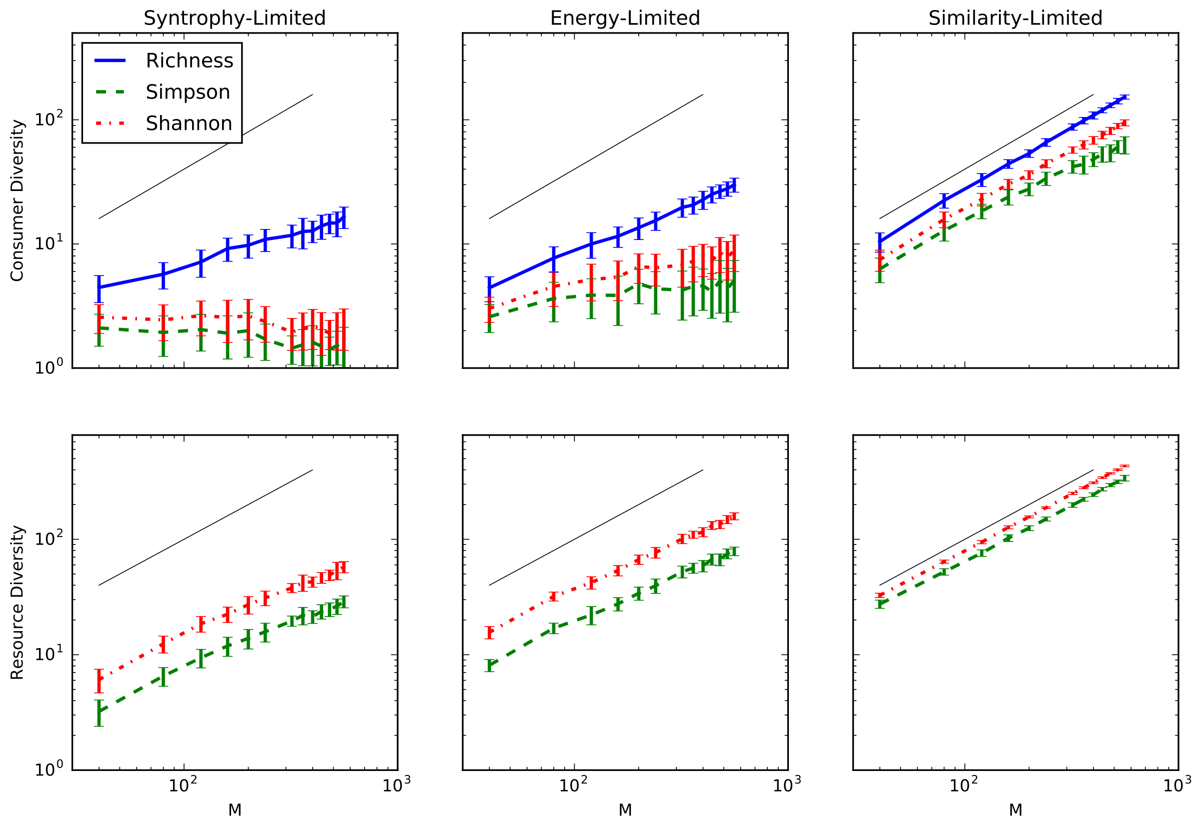

Figure S9: **Scaling of other observables.** Diversity of steady-state consumer and resource abundances in the three examples highlighted in Figure 2, as a function of the number of resource types M (with the rest of the parameters scaled as described in Supporting Text Section 1). The thin black line shows the slope corresponding to a linear scaling with M. Error bars are standard deviations over 100 samples per point.
