# Supplementary material for: Available energy fluxes drive a transition in the diversity, stability, and functional structure of microbial communities
Source: PLoS Comput Biol. 2019 Feb 5;15(2):e1006793. doi: 10.1371/journal.pcbi.1006793 (PMC6386421; doi:10.1371/journal.pcbi.1006793)
Supplement: S10 Fig — (PDF) [file pcbi.1006793.s011.pdf]

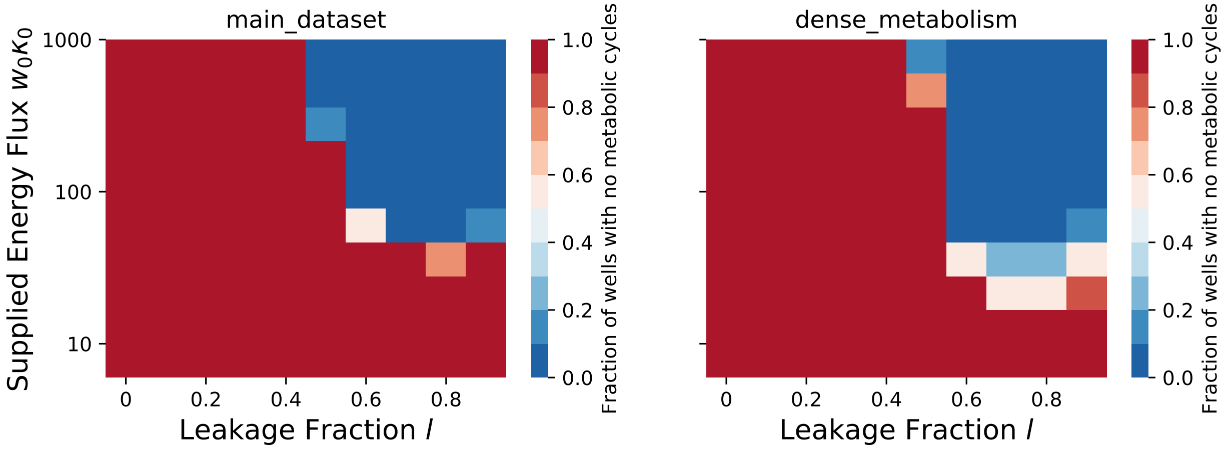

Figure S10: **Flux network topology.** We generated flux graphs like those of Figure 3 for both the sparse ('main_dataset') and the dense ('dense_metabolism') metabolic matrices shown there. We pruned the graphs as in the main text figure by removing edges with flux less than 1% of maximum flux in the network. For each condition, we used the Python package NetworkX (networkx.github.io) to determine how many of the ten replicates had acyclic graphs. The absence of cycles guarantees that these graphs can be topologically ordered like the left-hand panels of the figure. The color of each square represents the fraction of acyclic networks for the given values of l and $w_0\kappa_0$.
