# Supplementary material for: Available energy fluxes drive a transition in the diversity, stability, and functional structure of microbial communities
Source: PLoS Comput Biol. 2019 Feb 5;15(2):e1006793. doi: 10.1371/journal.pcbi.1006793 (PMC6386421; doi:10.1371/journal.pcbi.1006793)
Supplement: S11 Fig — (PDF) [file pcbi.1006793.s012.pdf]

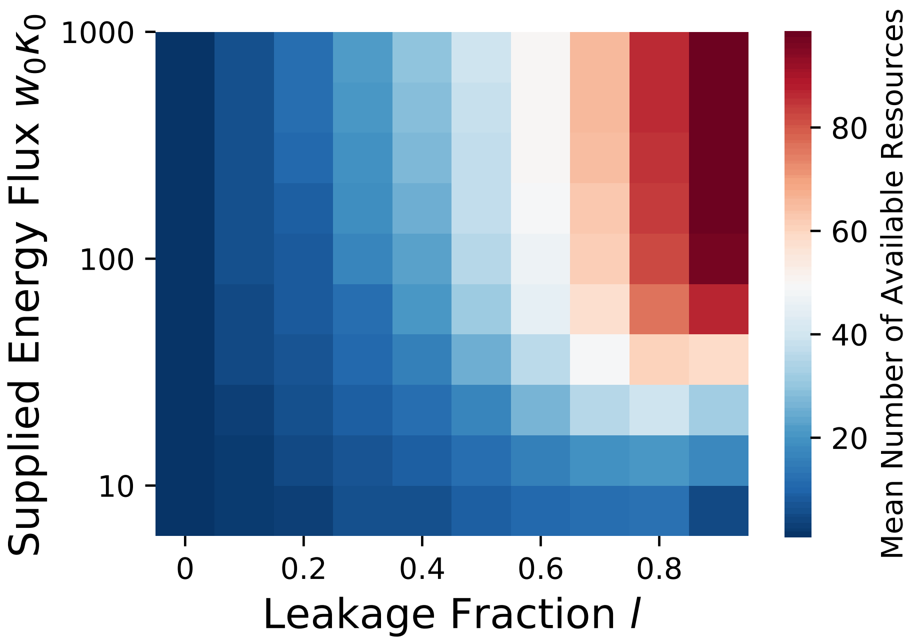

Figure S11: **Number of available resources.** The mean number of resources (over all ten initial communities) with abundances R_α above 0.1 is plotted for each of the conditions summarized in Figure 2. This threshold is the level at which an average consumer equally consuming 10 resources would be able to satisfy its full maintenance cost.
