# Supplementary material for: Available energy fluxes drive a transition in the diversity, stability, and functional structure of microbial communities
Source: PLoS Comput Biol. 2019 Feb 5;15(2):e1006793. doi: 10.1371/journal.pcbi.1006793 (PMC6386421; doi:10.1371/journal.pcbi.1006793)
Supplement: S12 Fig — (PDF) [file pcbi.1006793.s013.pdf]

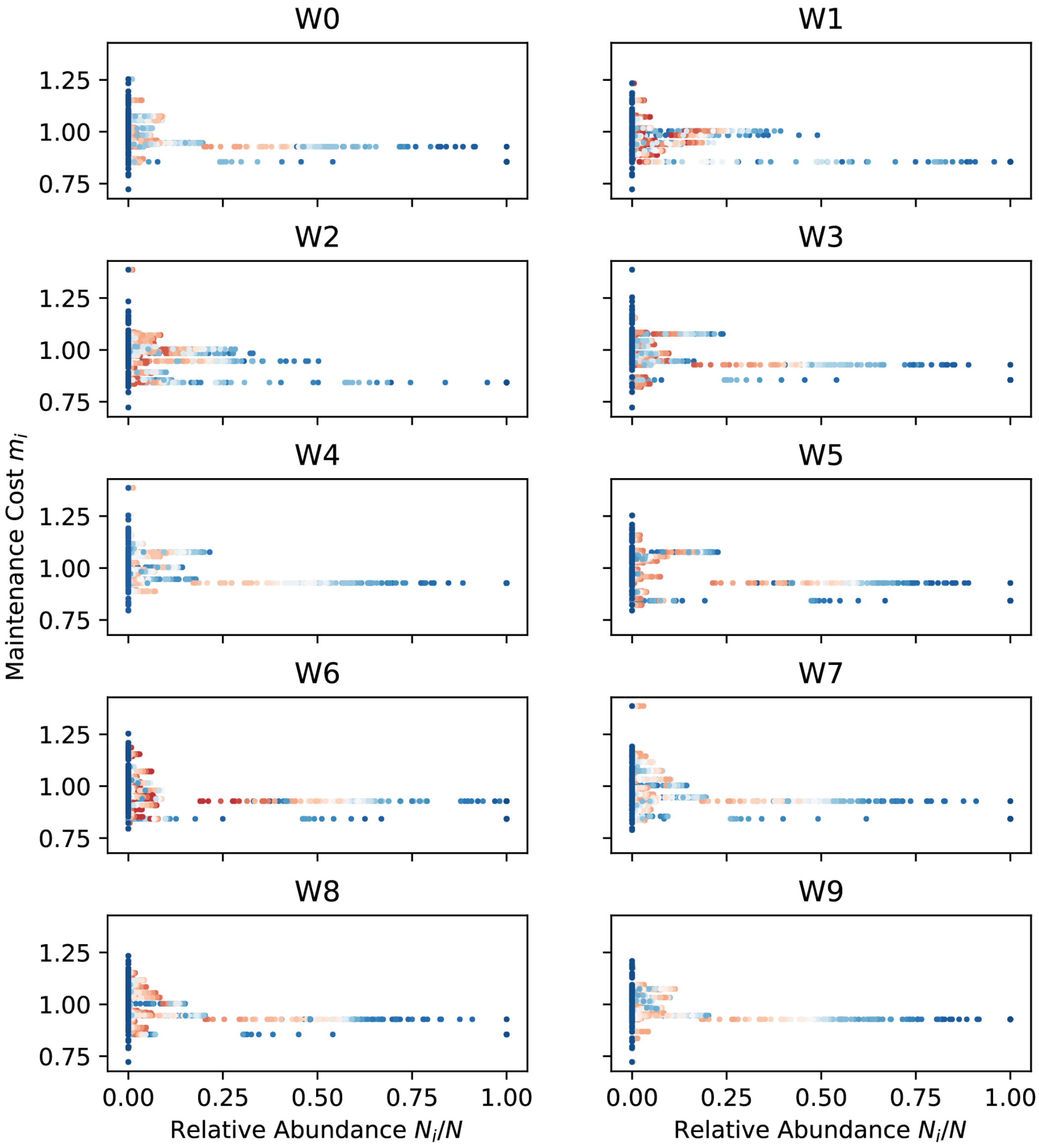


Figure S12: **Maintenance costs $m_i$.** The maintenance cost $m_i$ for each species is plotted against the relative abundance of that species in its community, for each of the simulations in Figure 2. The ten panels contain data for each of the ten initial pools of species. In each panel, each species has 100 data points, one for each of the 100 combinations of $l$ and $w_0\kappa_0$. The points are colored by the richness of the steady-state community, with blue being least diverse and red the most diverse. Every point in a given panel corresponds to a species that was initially present in the community, so points on the $m_i$ axis where all the relative abundances vanish are species that never survive to steady state in any of the sampled conditions.
